# Supplementary material for: Beyond network centrality: individual-level behavioral traits for predicting information superspreaders in social media
Source: Natl Sci Rev. 2024 Mar 1;11(7):nwae073. doi: 10.1093/nsr/nwae073 (PMC11173202; doi:10.1093/nsr/nwae073)
Supplement: nwae073_Supplemental_File [file nwae073_supplemental_file.pdf]

Beyond network centrality: Individual-level behavioral traits for predicting information superspreaders in social media

Supplementary Material

Fang Zhou[1,3], Linyuan Lü[2,1,*], Jianguo Liu[4], and Manuel Sebastian Mariani[1,5,*]

Contents

1. **Supplementary Tables** 2

2. **Supplementary Note 1: Validation in synthetic spreading data.** 4

3. **Supplementary Note 2: Robustness with respect to incomplete data.** 4

4. **Supplementary Note 3: Verifying that the empirical IS scores are consistent with the empirical results by Aral and Walker [1]** 9

5. **Supplementary Note 4: Statistical significance** 9

6. **Supplementary Note 5: Convergence conditions for the IS algorithm** 10
   - Theoretical analysis 10
   - Numerical analysis 12
   - Analytic convergence condition in directed network 15

7. **Supplementary Note 6: Numeric results on the convergence of the IS algorithm** 15

8. **Supplementary Note 7: Linear regression analysis** 18
   - 8.1. Variance inflation factor 18
   - 8.2. Linear mixed effect model 21

9. **Supplementary Note 8: Classification algorithms** 23
   - Random forest algorithm 23
   - XGBoost 23
   - Multilayer Perceptron 24

10. **Supplementary Note 9: Data collection, sampling, and partition** 24
    - Data collection 24
    - Data sampling 24
    - Data partition 24

11. **Supplementary Note 10: IS algorithm discussion and extension** 25

12. **Supplementary Figures** 25

---

[1] Institute of Fundamental and Frontier Sciences, University of Electronic Science and Technology of China, Chengdu 610054, P. R. China
[2] School of Cyber Science and Technology, University of Science and Technology of China, Hefei, 230026, P. R. China
[3] Yangtze Delta Region Institute (Huzhou), University of Electronic Science and Technology of China, Huzhou 313001, P. R. China
[4] Department of Digital Economics, Shanghai University of Finance and Economics, 200433, P. R. China
[5] URPP Social Networks, Universität Zürich, Switzerland
[*] e-mail:linyuan.lv@uestc.edu.cn; manuel.mariani@business.uzh.ch

1. Supplementary Tables

datasets	N	L	$\langle k \rangle$	σ	URL
Email	$1,133$	$5,451$	9.6	0.078	`https://github.com/zervel3/small-scale-social-datasets`
Facebook	$2,888$	$2,981$	2.1	-0.668	`https://github.com/zervel3/small-scale-social-datasets`
HepTh	$9,875$	$25,973$	5.3	0.268	`https://github.com/zervel3/small-scale-social-datasets`
Hamster	$1,858$	$12,534$	13.5	-0.080	`https://github.com/zervel3/small-scale-social-datasets`

Table S1 Details of the four small-scale empirical networks. We use four small-scale datasets with synthetic spreading events to validate the IS algorithm's performance, the Table shows the details of the four datasets, N is the size of the network, L is the sum of edges, $\langle k \rangle$ is the average degree, σ is the degree assortativity, the four datasets can be downloaded from the URL.

datasets	window 1	window 2	window 3	window 4	window 5	window 6
Weibo 2012	0.033	0.034	0.034	0.039	0.028	0.033
Weibo COVID	0.036	0.036	0.032	0.033	0.037	0.036
Twitter 2011	0	0.0	0.0	0	0.0	0.0
Twitter COVID	0.024	0.022	0.022	0.022	0.023	0.022

Table S2 Fraction of reconstructed edge probability that large than 1 in the 6 windows of 4 large-scale empirical datasets.

datasets	*Email*	*Facebook*	*HepTh*	*Hamster*
Influence	0.0	0.001	0.003	0.001
Susceptibility	0.001	0.002	0.002	0.002
Contagion probability	0.003	0.0	0.0	0.0

Table S3 Fraction of reconstructed Influence and susceptibility scores that are large than 1 in the 4 small-scale datasets.

Random forest model	Features	Datasets
Structure-based (Diffusion)	outdegree, indegree, PageRank, k-core	*Weibo COVID*, *Twitter COVID*
Behavioral-based (IS)	influence, susceptibility, total susceptibility, IS total score, total influence	*Weibo COVID*, *Twitter COVID*
Behavioral-based (CR)	total in-contagion rate, total out-contagion rate	*Weibo COVID*, *Twitter COVID*
Combined model	outdegree, indegree, PageRank, k-core, influence, susceptibility, total susceptibility, IS total score, total influence, past success	*Weibo COVID*, *Twitter COVID*
Structure-based (Follower)	outdegree, indegree	*Twitter COVID*

Table S4 The random forest models and corresponding features.

2. Supplementary Note 1: Validation in synthetic spreading data.

To test the IS algorithm's performance in reconstructing the individual influence and susceptibility, based on the known ground-truth influence and susceptibility scores, we simulate spreading processes with the empirical influence model on a given network to obtain its counterpart diffusion network. Specifically, we start by drawing, for each individual i, the ground-truth influence and susceptibility scores, I_i and S_i, from a uniform distribution between 0 and 1; these scores determine the ground-truth contagion probability between any two individuals i and j: $p_{ij} = A_{ij}\, I_i\, S_j$. We simulate $100\,N$ spreading processes: each individual $i \in \{1, \dots, N\}$ is the seed individual of 100 cascades. In a simulated spreading process, at each time step, an individual i who already reshared the piece of information ($r_i = 1$) attempts once to propagate the information to each of her neighbors to reshare; such a propagation event has a probability p_{ij}. Each spreading process stops when no individuals can be influenced by their neighbors. For simplicity, we simulate the spreading on four small-scale empirical networks used in prior works [2–4] (see Table S3 for details). After having simulated the spreading process, a diffusion network is constructed for each dataset, where the IS algorithm can be applied to reconstruct the influence ($\hat{I}$) and susceptibility ($\hat{S}$) scores of all individuals.

Datasets	$r(\hat{I}, I)$	$r(\hat{I}, f)$	$r(\hat{I}, k)$	$r(\hat{S}, S)$	$r(\hat{S}, g)$	$r(\hat{S}, k)$
Email	$\mathbf{0.999}$	0.468	0.040	$\mathbf{1.000}$	0.434	-0.021
Facebook	$\mathbf{0.791}$	0.147	0.120	$\mathbf{0.912}$	0.135	0.095
HepTh	$\mathbf{0.970}$	0.388	0.016	$\mathbf{0.968}$	0.391	0.011
Hamster	$\mathbf{0.982}$	0.327	0.018	$\mathbf{0.986}$	0.308	0.003

Table S5 IS algorithm's performance on four small-scale networks, based on synthetic spreading events. I, f, S, g, k denote individuals' ground-truth influence, outgoing contagion rate, susceptibility, incoming contagion rate, and degree, respectively. $\hat{I}$ and $\hat{S}$ denote the reconstructed influence and susceptibility, respectively. $r(a, b)$ denotes the Pearson's correlation coefficient between a given pair (a, b) of variables.

Table S3 illustrates the reconstruction accuracy of the IS algorithm on the four small-scale networks, which reveals three key insights. First, the results show that degree is uncorrelated with reconstructed influence ($\hat{I}$), indicating that in this setting, the social hubs are not guaranteed to be highly influential. Second, although the ground-truth outgoing contagion rate f is positively correlated with the reconstructed influence, the linear correlation is moderate at best – ranging from $r = 0.15$ in *Facebook* to $r = 0.47$ in *Email*. Third, the reconstructed influence derived by IS algorithm achieves a remarkably high correlation with the ground-truth influence, ranging from 0.79 in *Facebook* to 1.00 in *Email*. Similar insights can be obtained for the reconstruction of susceptibility (see Figs. S11, S12, S13, S14 for the density plots of the four small-scale datasets). Taken together, these observations indicate that in synthetic spreading data, the IS algorithm effectively reconstructs the ground-truth influence and susceptibility scores.

3. Supplementary Note 2: Robustness with respect to incomplete data.

In real-world applications, we may have only access to data on a limited number of spreading events. To ascertain the robustness of the algorithm's performance with respect to incomplete data, we study the performance of the algorithm as we progressively remove randomly selected spreading events from the generated synthetic data.

Specifically, on the four small-scale datasets, we set each individual as seed node and run 5 times Independent Cascade spreading process [5] to get a series of $(5N)$ spreading events, and calculate the edge weight p_{ij} by the whole spreading events, where p_{ij} is the fraction of tweets that j retweets from i, then we input the p_{ij} and network structure to IS algorithm to reconstruct the I and S and take them as the baseline, then remove a fraction of spreading events, calculate the edge weight $\hat{p}_{ij}$ by remaining spreading events, finally, estimate individuals $\hat{I}$ and $\hat{S}$, here we use the Pearson's correlation r and mean absolute error (MAE) to measure how the IS algorithm performs when the fraction of spreading events are removed. The results are averaged by 10 realizations.

We find that the accuracy of the IS algorithm in reconstructing the ground-truth influence and susceptibility scores decrease linearly with the number of removed spreading events (see Figs. S1, S2, S3, S4 for details). However, a relatively high correlation can be maintained even if a substantial portion of the spreading events is removed. For example, when 30% spreading events are removed from $Email$, the scores produced by the IS algorithm still exhibit a high correlation with the ground-truth influence and susceptibility scores ($r = 0.69$ and $r = 0.83$, respectively; see Fig. S1 for details). These results indicate that the IS algorithm can not only accurately reconstruct ground-truth influence and susceptibility scores, but also has remarkable robustness against data incompleteness.

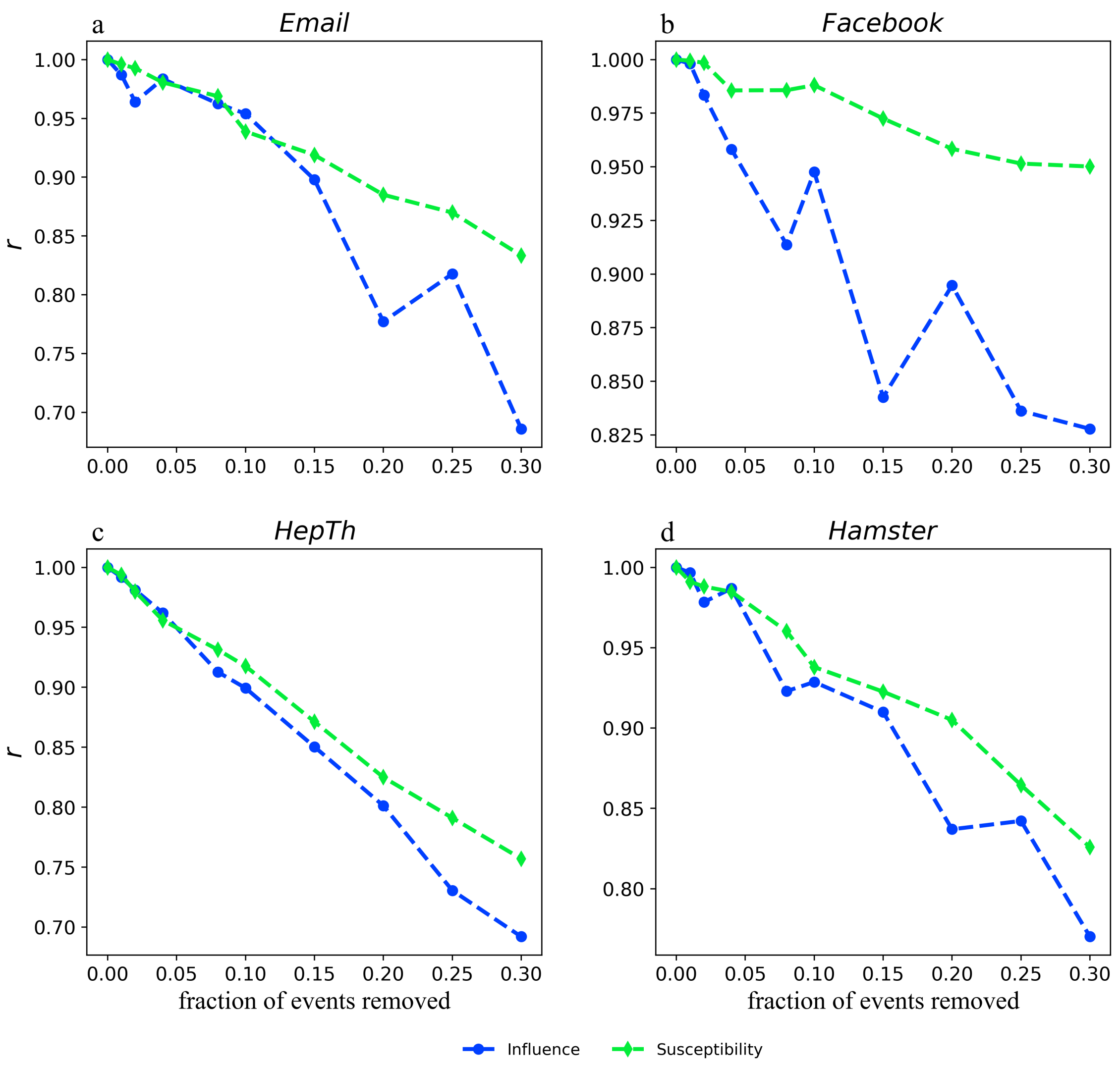

Fig. S1 Robustness of IS algorithm measured by Pearson's coefficient. The x-axis denotes the fraction of spreading events removed, ranging from 0.01 to 0.3, r is the Pearson's coefficient between the original I (S) and the $\hat{I}$ ($\hat{S}$) estimated by the incomplete spreading events.

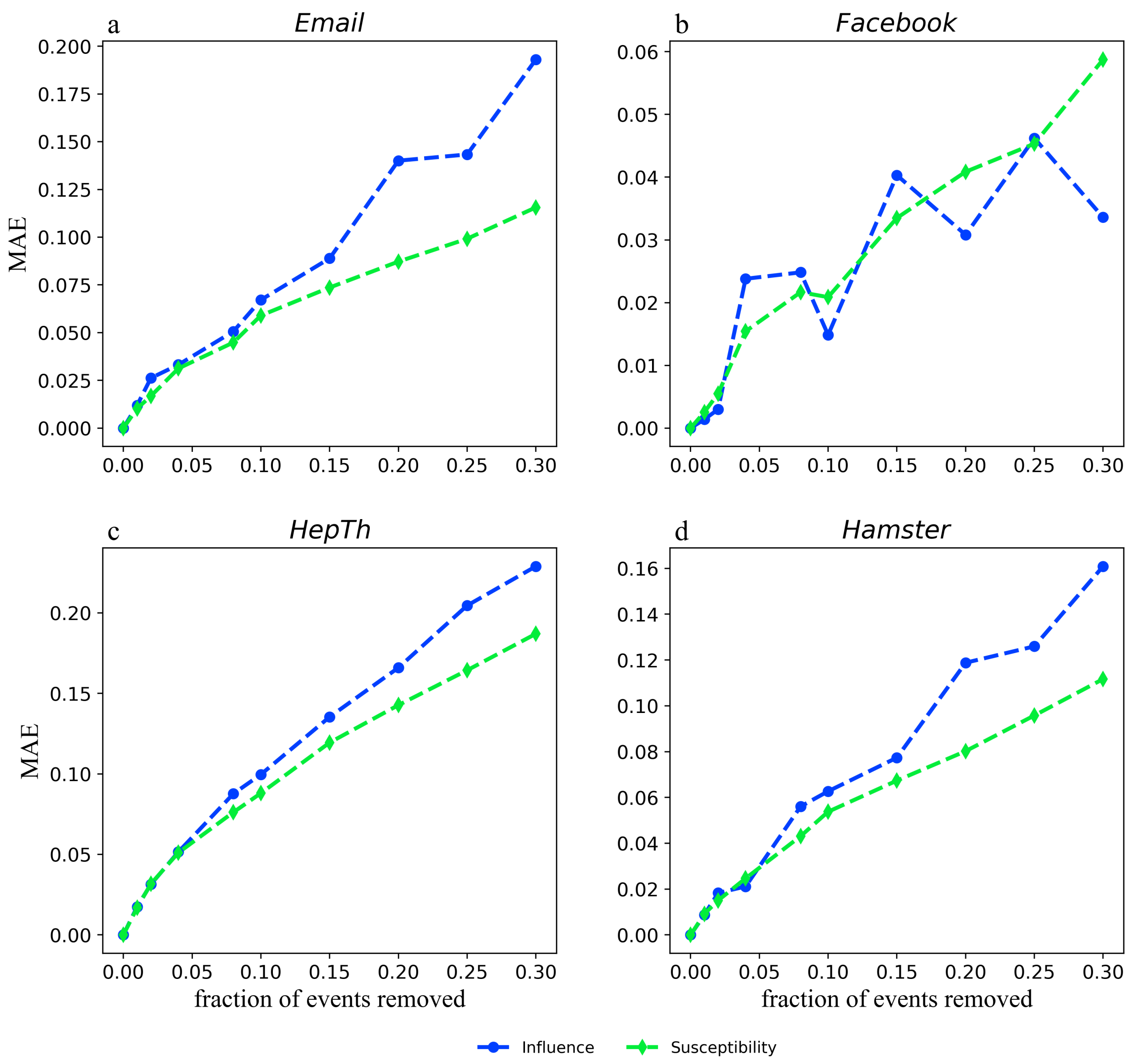

Fig. S2 Robustness of IS algorithm measured by mean absolute error. The x-axis denotes the fraction of spreading events removed, ranging from 0.01 to 0.3, MAE is the mean absolute error between the original I (S) and the $\hat{I}$ $(\hat{S})$ estimated by the incomplete spreading events.

We also applied to the four large-scale empirical datasets to validate the robustness of the IS algorithm, specifically, we set the I and S scores reconstructed by complete spreading events as the baseline, and randomly remove the fraction of retweets (spreading events), then calculate the Pearson's correlation and mean absolute error between the baseline and the scores ($\hat{I}$ and $\hat{S}$) reconstructed by incomplete spreading events.

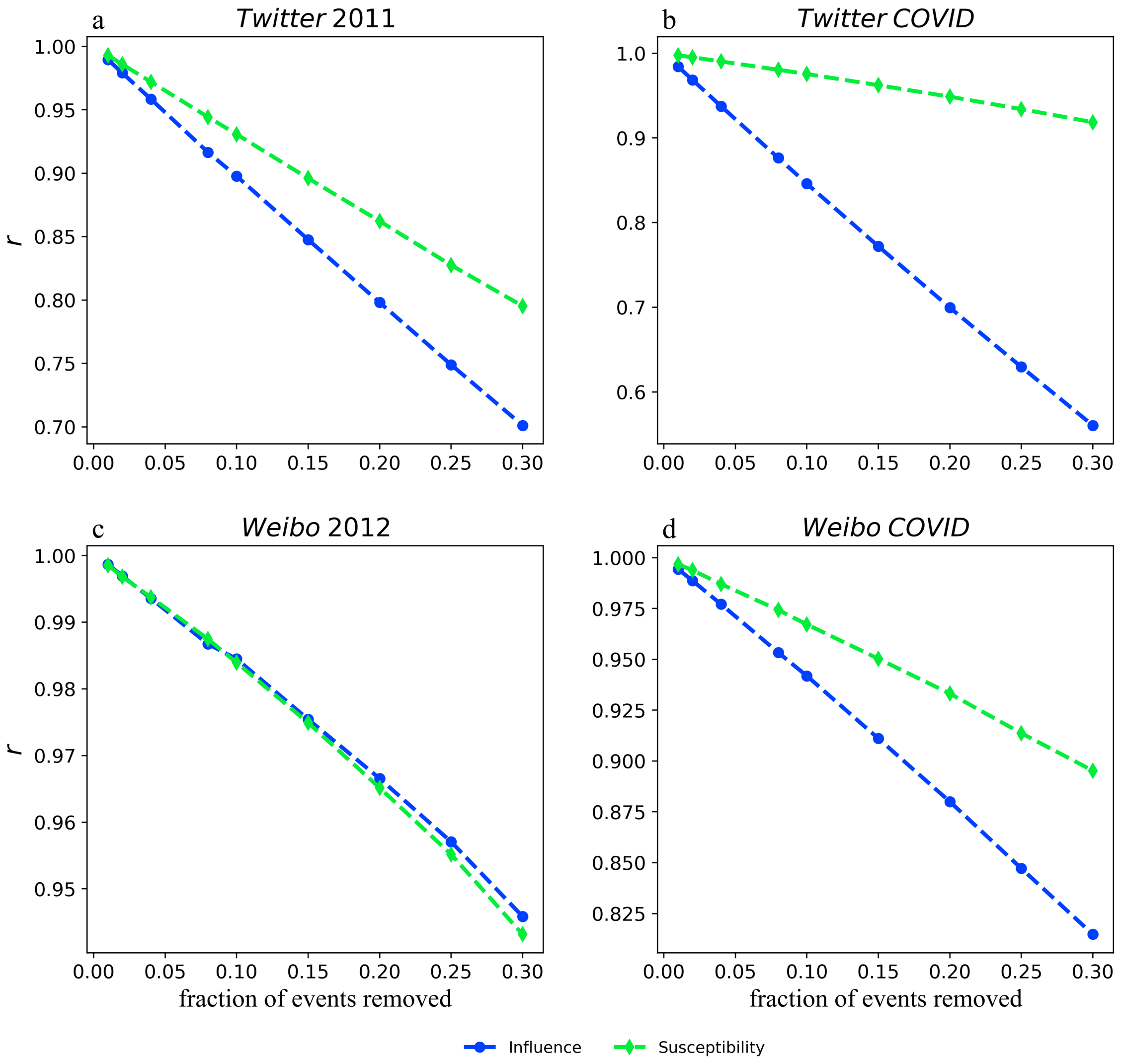

Fig. S3 Robustness of IS algorithm measured by Pearson's coefficient. The x-axis denotes the fraction of spreading events removed, r is the Pearson's coefficient between the original I (S) and the $\hat{I}$ ($\hat{S}$) estimated by the incomplete spreading events.

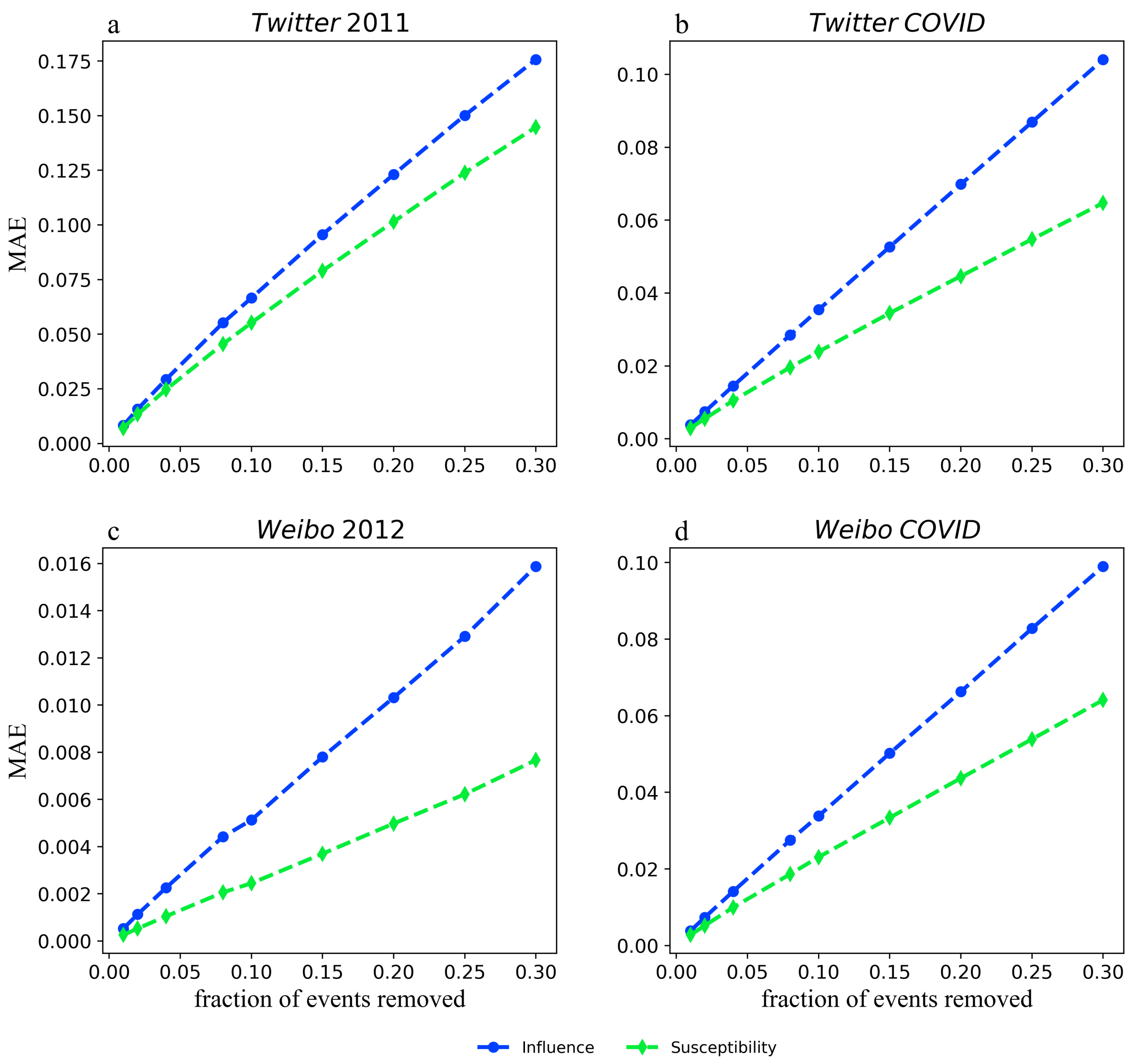

Fig. S4 Robustness of IS algorithm measured by mean absolute error. The x-axis denotes the fraction of spreading events removed, MAE is the mean absolute error between the original I (S) and the $\hat{I}$ ($\hat{S}$) estimated by the incomplete spreading events.

4. Supplementary Note 3: Verifying that the empirical IS scores are consistent with the empirical results by Aral and Walker [1]

Validating the algorithm in empirical spreading data faces the natural challenge that we lack access to the individuals' ground-truth influence and susceptibility scores. To bypass this challenge, we only validate the aggregate properties of the scores. Specifically, we verify whether, when applied to empirical spreading data from social media, the algorithm produces scores that respect the four stylized facts about influence and susceptibility identified in Aral and Walker's experiment [1]: (i) highly influential individuals tend not to be susceptible, highly susceptible individuals tend not to be influential, and almost no one is both highly influential and highly susceptible to influence; (ii) both influential individuals and non-influential individuals have approximately the same distribution of susceptibility to influence among their peers; (iii) there are more people with high influence scores than high susceptibility scores; (iv) influentials cluster in the network.

To this end, we apply the algorithm to four large-scale empirical datasets: *Twitter 2011*, *Twitter COVID*, *Weibo 2012*, and *Weibo COVID*. In empirical social networks, the diffusion network is constructed from the spreading events among users, which is different from the social networks that connect followers and followees [6]. For this reason, we run the IS algorithm on the diffusion network [6] whose directed adjacency matrix's element $A_{ij} = 1$ if i retweeted at least once from j. With this choice, i's indegree $k^{in} = \sum_j A_{ji}$ denotes the number of individuals i retweeted from; her outdegree $k^{out} = \sum_j A_{ij}$ denotes the number of individuals who retweeted from i. Remarkably, for all the four analyzed large-scale empirical datasets, the above-mentioned stylized facts (i–iii) identified by Aral and Walker [1] hold, whereas the validity of stylized fact (iv) is dataset-dependent. This indicates that in empirical spreading data, the algorithm produces scores that respect the properties we would expect for influence and susceptibility scores from the experiment [1].

First, we test whether **highly influential individuals tend not to be susceptible, highly susceptible individuals tend not to be influential, and almost no one is both highly influential and highly susceptible to influence.** For the first two claims, it's proved by the negative and low correlation between I and S for *Twitter 2011*, *Twitter COVID*, *Weibo 2012* and *Weibo COVID*, they are $r(I,S) = -0.56$, $r(I,S) = -0.11$, $r(I,S) = -0.09$ and $r(I,S) = -0.52$ respectively, for the third claim, we choose top 0.5% of individuals by their influence and susceptibility score respectively, for *Twitter 2011*, *Twitter COVID*, *Weibo 2012* and *Weibo COVID*, the overlap of the above two types of individuals are 0.2%, 0.3%, 0.9% and 0.1%, such a value is very low. When we increase the fraction of individuals chosen to top 1%, the overlap are 0.5%, 0.5%, 1.5%, 2% respectively.

Second, we test whether **both influential individuals and noninfluential individuals have approximately the same distribution of susceptibility to influence among their peers.** To test this section, we rank individuals by their influence score, and choose the top 0.5% of influentials' (individuals with high influence score) susceptibility score, then do the Mann-Whitney test between influentials susceptibility score and remaining individuals' susceptibility score, the result shows, for *Twitter 2011*, *Twitter COVID*, *Weibo 2012* and *Weibo COVID*, their influentials' susceptibility score and remaining 99.5% individuals' susceptibility score are from the same distribution with the probability 99%.

Third, we test whether **there are more people with high influence scores than high susceptibility scores.** We need to define what having a high score means. We make a conservative choice, and consider an influence score $\hat{I}$ as high if and only if $\hat{I} > \mu_I + 3\,\sigma_I$, where μ_I and σ_I are the mean and standard deviation of the influence score across the whole population. Analogously, we consider a susceptibility score $\hat{S}$ as high if and only if $\hat{S} > \mu_S + 3\,\sigma_S$, where μ_S and σ_S are the mean and standard deviation of the susceptibility score across the whole population. We denote the fraction of high influence score and high susceptibility score individuals as F_{inf} and F_{sus}, respectively. For *Twitter 2011*, the F_{inf} and F_{sus} are 0.45% and 0.4%. For *Twitter COVID*, the F_{inf} and F_{sus} are 2.7%, 1.8%, respectively. For *Weibo 2012*, the F_{inf} and F_{sus} are 2.0%, 0.1%, respectively. For *Weibo COVID*, under the condition $\hat{I} > \mu_I + 2\,\sigma_I$ and $\hat{S} > \mu_S + 2\,\sigma_S$, the F_{inf} and F_{sus} are 4.3%, 1.9% respectively.

Finally, we test whether **influentials cluster in the network.** We choose the top 0.5% individuals by their influence score as influentials, for *Twitter 2011*, we find 1.6% of influentials have at least an edge to other influentials, which is significantly larger than the same fraction in networks randomized with the directed configuration model is $0.51\% + 0.11\%$. For *Twitter COVID*, 1.98% fraction of influentials have at least an edge to other influentials, the corresponding value in the directed configuration model is $1.12\% \pm 0.07\%$. For *Weibo 2012*, 6.41% of influentials have at least an edge to other influentials, and the corresponding value in the directed configuration model is $5.73\% \pm 0.31\%$. But for *Weibo COVID*, 0.06% of influentials have at least an edge to other influentials, the corresponding value in the directed configuration model is $0.14\% \pm 0.01\%$.

5. Supplementary Note 4: Statistical significance

To assess the statistical significance of Spearman's correlations between individuals' properties, we randomize the edge weights of the whole diffusion network by drawing each edge weight from the uniform distribution in $[0, 1]$. Based on this randomized network, we estimate the individuals' properties with the IS algorithm and compare the original Spearman's values with the values obtained in the randomized networks. For each dataset, 20 independent realizations of this process are performed.

For each pair of properties (e.g., $\rho(I,S)$), we define the P-value as the fraction of realizations where the correlation is larger or smaller than the original correlation.

To test the statistical significance of the assortativity between individuals' properties and their neighbors' properties, we use the directed configuration model[7] to randomize the network structure. The model randomly rewires the edges while preserving the given degree sequences. To analyze whether the network structure influences the assortativity properties, for each network, we keep the original I and S scores fixed, obtain 20 randomized networks with the directed configuration model, and compare the original assortativity properties with those in the rewired networks. For each pair of properties, we define the P-value as the fraction of randomized networks where the correlation is larger or smaller than the original correlation.

6. Supplementary Note 5: Convergence conditions for the IS algorithm

Theoretical analysis

Conclusion 1: When starting with identical and non-negative initial values for the influence and susceptibility scores, if the network is not a tree and not a complete network, the IS algorithm will converge to a unique solution. The proof process is below.

Part of the following proof borrows the idea from paper [8], we find a sufficient and unnecessary condition that guarantees the convergence of equations (5) in the main text. Specifically, We convert equations (3) and (4) in the main text into a fixed point function $x = G(x)$. By denoting the Jacobian matrix of $G(x)$ as $\mathbf{J}$, we prove the fixed point equation $x = G(x)$ converges to a stable solution when $\rho(\mathbf{J}) < 1$, where $\rho(\mathbf{J})$ is the spectral radius of matrix $\mathbf{J}$. The main result is that for both undirected and directed networks, the algorithm converges when the initial values $I^{(0)}$ and $S^{(0)}$ are both identical non-negative values.

Following is the proof process. Since equations (5) in the main text are non-linear equations, to prove their convergence, we can convert equations (3), and (4) in the main text to the fixed-point problem, with the type $x = G(x)$.

For the following two equations (Equations (3), and (4) in the main text)

$$I_i = \frac{f_i}{\sum_j A_{ij} S_j} \tag{1}$$

$$S_j = \frac{g_j}{\sum_i A_{ij} I_i} \tag{2}$$

We set $I_i = x_i^{-1}$, $S_j = y_j^{-1}$. The above two equations are transformed as

$$x_i = \frac{\sum_j A_{ij} y_j^{-1}}{f_i} \tag{3}$$

$$y_j = \frac{\sum_i A_{ij} x_i^{-1}}{g_j} \tag{4}$$

Following we will construct a block matrix with the form $x = G(x)$. After applying equations (3) and (4) to the whole network, we get the following $2N$-dimensional (N is the size of the network) vector equations.

$$\begin{pmatrix} x_1 \\ \vdots \\ x_N \\ y_1 \\ \vdots \\ y_N \end{pmatrix} = \begin{pmatrix} \mathbf{0} & \mathbf{FA} \\ \mathbf{EA^T} & \mathbf{0} \end{pmatrix} \begin{pmatrix} x_1^{-1} \\ \vdots \\ x_N^{-1} \\ y_1^{-1} \\ \vdots \\ y_N^{-1} \end{pmatrix} \tag{5}$$

Where

$$\mathbf{F} = \begin{pmatrix} \frac{1}{f_1} & 0 & \cdots & 0 \\ 0 & \frac{1}{f_2} & \cdots & 0 \\ \vdots & \vdots & \ddots & \vdots \\ 0 & 0 & \cdots & \frac{1}{f_N} \end{pmatrix}, \mathbf{E} = \begin{pmatrix} \frac{1}{g_1} & 0 & \cdots & 0 \\ 0 & \frac{1}{g_2} & \cdots & 0 \\ \vdots & \vdots & \ddots & \vdots \\ 0 & 0 & \cdots & \frac{1}{g_N} \end{pmatrix}$$

$\mathbf{A}$ is the adjacent matrix. The equation (5) with the type $x = G(x)$ is what we need, it can be seen as a fixed-point iterative problem, and to prove its convergence, we need to prove at one given initial condition x^*, the spectral radius of its Jacobian matrix is small than 1, that is $\rho(\frac{\partial G(x^*)}{\partial x}) < 1$.

Following we will calculate the Jacobian matrix of $G(x)$.

$$\mathbf{J} = \frac{\partial G(x)}{\partial x} = [\frac{\partial G(x)}{\partial x_1}, \cdots, \frac{\partial G(x)}{\partial x_N}, \frac{\partial G(x)}{\partial y_1}, \cdots, \frac{\partial G(x)}{\partial y_N}] \tag{6}$$

The $G(x)$ can be written as $G(x) = \mathbf{M}v(x)$, where

$$\mathbf{M} = \begin{pmatrix} \mathbf{0} & \mathbf{FA} \\ \mathbf{EA^T} & \mathbf{0} \end{pmatrix}, v(x) = \begin{pmatrix} x_1^{-1} \\ \vdots \\ x_N^{-1} \\ y_1^{-1} \\ \vdots \\ y_N^{-1} \end{pmatrix}$$

$\mathbf{M}$ is a constant matrix, so we only need to calculate the derivative of $v(x)$, finally we got

$$\mathbf{J} = \frac{\partial G(x)}{\partial x} = \begin{pmatrix} \mathbf{0} & -\mathbf{FAY^{-2}} \\ -\mathbf{EA^TX^{-2}} & \mathbf{0} \end{pmatrix} \tag{7}$$

$$\mathbf{Y^{-2}} = \begin{pmatrix} y_1^{-2} & 0 & \cdots & 0 \\ 0 & y_2^{-2} & \cdots & 0 \\ \vdots & \vdots & \ddots & \vdots \\ 0 & 0 & \cdots & y_N^{-2} \end{pmatrix}, \mathbf{X^{-2}} = \begin{pmatrix} x_1^{-2} & 0 & \cdots & 0 \\ 0 & x_2^{-2} & \cdots & 0 \\ \vdots & \vdots & \ddots & \vdots \\ 0 & 0 & \cdots & x_N^{-2} \end{pmatrix}$$

It is hard to calculate the spectral radius of $\mathbf{J}$ directly when applied to a large-scale network. We turn to estimate its spectral radius. The square of $\mathbf{J}$ can be written as

$$\mathbf{J^2} = \begin{pmatrix} \mathbf{FAY^{-2}EA^TX^{-2}} & \mathbf{0} \\ \mathbf{0} & \mathbf{EA^TX^{-2}FAY^{-2}} \end{pmatrix} \tag{8}$$

We noticed that the two blocks $\mathbf{FAY^{-2}EA^TX^{-2}}$ and $\mathbf{EA^TX^{-2}FAY^{-2}}$ with same eigenvalues.

$\mathbf{FAY^{-2}EA^TX^{-2}}$ is

$$\begin{pmatrix} \frac{1}{f_1x_1^2}\sum_j A_{1j}A_{1j}\frac{1}{g_jy_j^2} & \frac{1}{f_1x_2^2}\sum_j A_{1j}A_{2j}\frac{1}{g_jy_j^2} & \cdots & \frac{1}{f_1x_N^2}\sum_j A_{1j}A_{Nj}\frac{1}{g_jy_j^2} \\ \frac{1}{f_2x_1^2}\sum_j A_{2j}A_{1j}\frac{1}{g_jy_j^2} & \frac{1}{f_2x_2^2}\sum_j A_{2j}A_{2j}\frac{1}{g_jy_j^2} & \cdots & \frac{1}{f_2x_N^2}\sum_j A_{2j}A_{Nj}\frac{1}{g_jy_j^2} \\ \vdots & \vdots & \ddots & \vdots \\ \frac{1}{f_Nx_1^2}\sum_j A_{Nj}A_{1j}\frac{1}{g_jy_j^2} & \frac{1}{f_Nx_2^2}\sum_j A_{Nj}A_{2j}\frac{1}{g_jy_j^2} & \cdots & \frac{1}{f_Nx_N^2}\sum_j A_{Nj}A_{Nj}\frac{1}{g_jy_j^2} \end{pmatrix} \tag{9}$$

By setting x_i^*, y_i^* both equal to non-negative I_0 as the initial condition, the above matrix is simplified as

$$\mathbf{Q} = \begin{pmatrix} \frac{1}{k_1}\sum_j A_{1j}A_{1j}\frac{1}{k_j} & \frac{1}{k_1}\sum_j A_{1j}A_{2j}\frac{1}{k_j} & \cdots & \frac{1}{k_1}\sum_j A_{1j}A_{Nj}\frac{1}{k_j} \\ \frac{1}{k_2}\sum_j A_{2j}A_{1j}\frac{1}{k_j} & \frac{1}{k_2}\sum_j A_{2j}A_{2j}\frac{1}{k_j} & \cdots & \frac{1}{k_2}\sum_j A_{2j}A_{Nj}\frac{1}{k_j} \\ \vdots & \vdots & \ddots & \vdots \\ \frac{1}{k_N}\sum_j A_{Nj}A_{1j}\frac{1}{k_j} & \frac{1}{k_N}\sum_j A_{Nj}A_{2j}\frac{1}{k_j} & \cdots & \frac{1}{k_N}\sum_j A_{Nj}A_{Nj}\frac{1}{k_j} \end{pmatrix} \tag{10}$$

We noticed that the matrix $\mathbf{J^2}$ is the block matrix, that is

$$\mathbf{J^2} = \begin{pmatrix} \mathbf{Q} & \mathbf{0} \\ \mathbf{0} & \mathbf{Q^T} \end{pmatrix} \tag{11}$$

To estimate the spectral radius of $\mathbf{J^2}$, we turn to estimate the spectral radius of $\mathbf{Q}$ by the Gershgorin circle theorem [9], which is equivalent to proving the modulo of the maximum eigenvalue (spectral radius) of $\mathbf{Q}$ is small than or equal to 1. According to the deduction of Gershgorin circle theorem, for the matrix $\mathbf{Q}$, $max_i|\lambda_i| \leq max_i(|q_{ii}| + \sum_{j=1,j\neq i}^{n}|q_{ij}|)$ (here q_{ij} is the

element of $\mathbf{Q}$), because the element of the matrix $\mathbf{Q}$ is always the positive real number, proving the spectral radius small than or equal to 1 is equivalent to prove the sum of arbitrary row's element is small than or equal to 1, which is to prove $\sum_{j=1}^{n} q_{ij} = \frac{1}{k_i}\sum_j \sum_l A_{ij}A_{lj}\frac{1}{k_j} \leq 1$ hold. When k_i is 1 and i's neighbor with degree 1, we get $\frac{1}{k_i}\sum_j \sum_l A_{ij}A_{lj}\frac{1}{k_j} = 1$ hold, otherwise, it's small than 1. When k_i is big than 1, to maximize $\sum_j \sum_l A_{ij}A_{lj}\frac{1}{k_j}$, we get two cases, first is all i's neighbors don't connected together and with degree 1, which means i's neighbor only connect with i, in such a case, we get $\sum_j \sum_l A_{ij}A_{lj}\frac{1}{k_j} = k_i$, second is all i's neighbors are fully connected (We can say its clustering coefficient $c_i = 1$), in such a case, $\sum_j \sum_l A_{ij}A_{lj}\frac{1}{k_j} = k_i$ as well, except for the above two cases, $\sum_j \sum_l A_{ij}A_{lj}\frac{1}{k_j} < k_i$ always hold, so we proved for matrix $\mathbf{Q}$, the sum of each row's elements small than or equal to 1 when we set x_i^* and y_i^* both equal to nonnegative I_0, according to the Gershgorin circle theorem [9], we know the spectral radius $\rho(\mathbf{Q}) \leq 1$, finally we proved $\rho(\mathbf{J}) \leq 1$. Besides, the case $\rho(\mathbf{J}) = 1$ holds if and only if for arbitrary i, its clustering coefficient c_i is either 0 or 1 (In this case, the sum of each row of matrix J^2 is 1, getting $\mathbf{J^2}$ with an eigenvalue 1, then $\rho(\mathbf{J}) = 1$), In other cases, we have $\rho(\mathbf{J}) < 1$. In conclusion, if the network's clustering coefficient is both not 0 and 1. Then, when we choose the same and non-negative initial values I^0 and S^0. The algorithm always converges to a unique solution.

Numerical analysis

Conclusion 2: When starting with non-negative initial values for the influence and susceptibility scores, in the studied cases (4 small datasets, 4 large-scale datasets, tree networks, and complete networks), we find numerically that if the network is not a tree and not a complete network, the IS algorithm will converge to a unique solution.

The conclusion 2 is given by simulation. Specifically, we randomly choose non-negative initial influence and susceptibility values (not required same) and start iterating the algorithm. We find the solutions of different realizations remain consistent. For 4 small-scale datasets, we run 1000 times with random initial values. For the 4 large-scale empirical datasets, we run 100 times. We present the influence and susceptibility scatter plots of 5 experiments of 2 small-scale datasets (*Email*, *HepTh*) and 2 large-scale empirical datasets (*Weibo* and *Weibo COVID*, only the first time window is chosen) in Figs. S5, S6. The results show that under non-negative different initial values, the solutions are the same.

Conclusion 3: When starting with non-negative initial values for the influence and susceptibility scores, if the network is a tree or a complete network, the IS algorithm will not converge to a unique solution.

To prove the conclusion 3 numerically, we choose two networks whose clustering coefficient is 0 or 1. They are the tree network and the complete network, to compare the results with a network that clustering is not 0 or 1, we also present the result of *Email* network. Then, we choose individuals' ground-truth influence and susceptibility by uniform distribution from 0 to 1 and give different initial values to iterate the IS algorithm. We present the scatter plots between the ground-truth influence (susceptibility) score and the score obtained from 4 experiments with different initial values, see Figs. S7 and S8 for the scatter plots of influence and susceptibility, respectively.

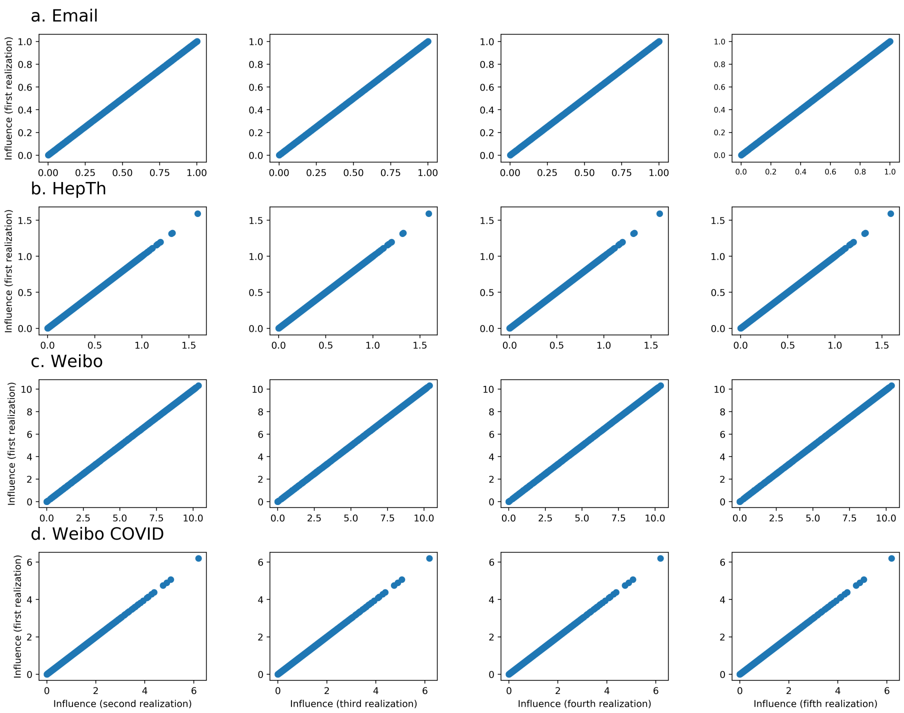

Fig. S5 Influence scores scatter plots of different realizations.

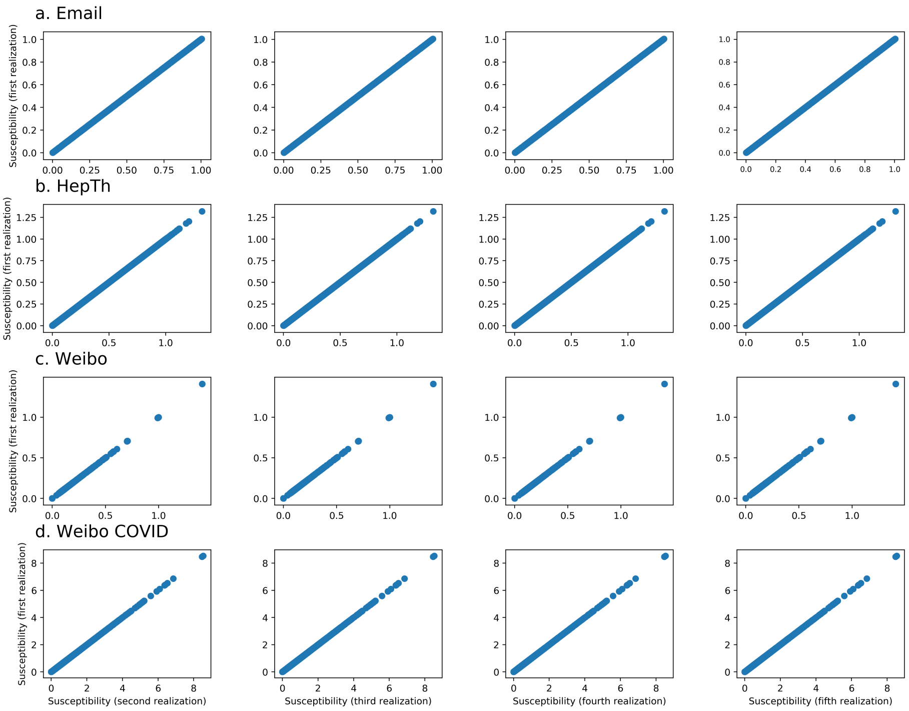

Fig. S6 Susceptibility scores scatter plots of different realizations.

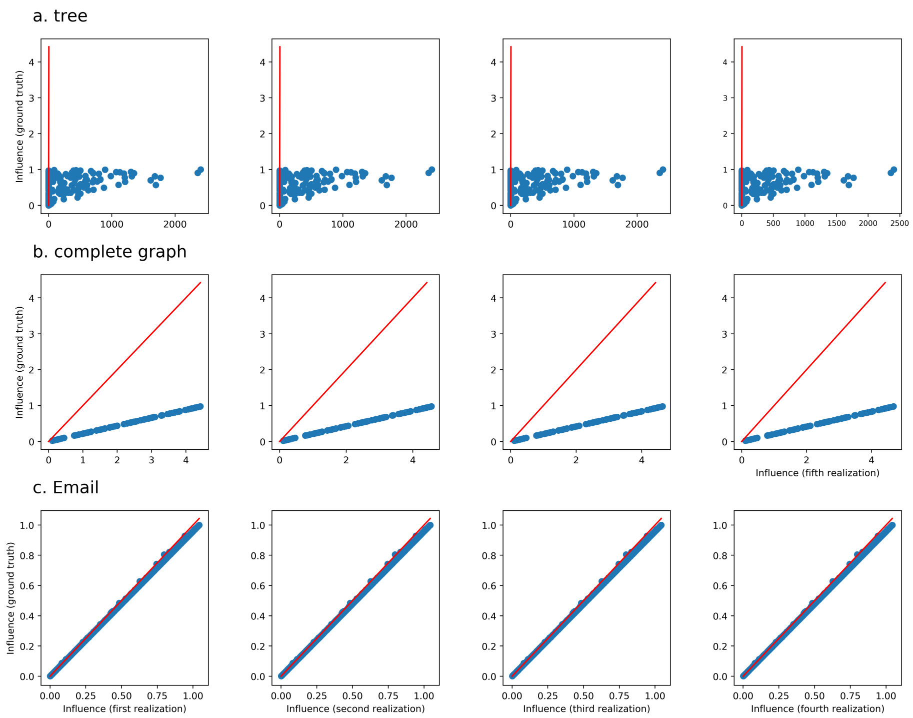

Fig. S7 Scatter plots between ground-truth influence scores and scores estimated from different realizations.

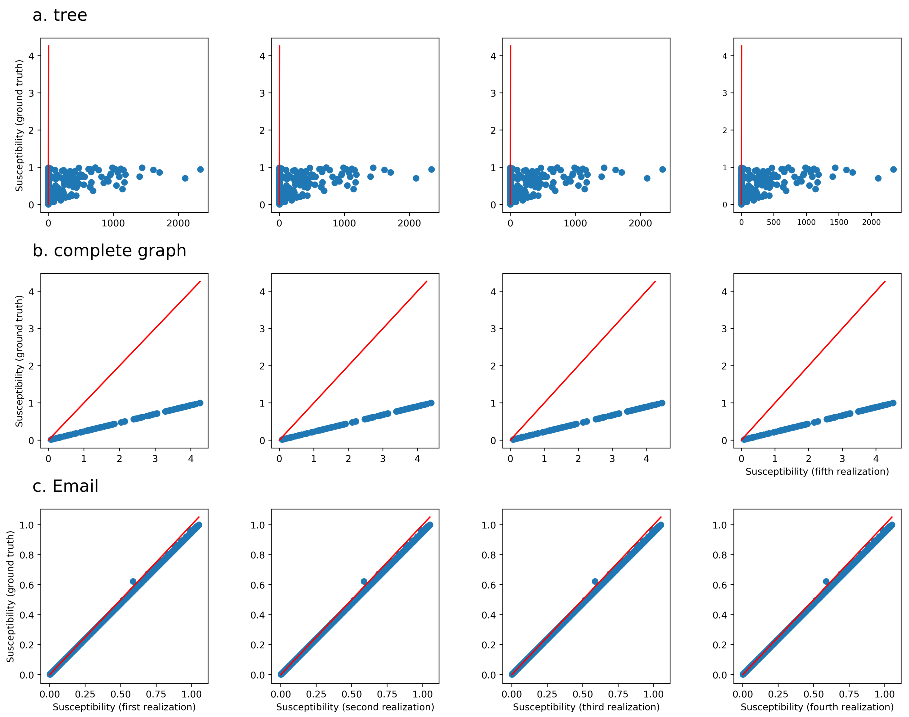

Fig. S8 Scatter plots between ground-truth susceptibility scores and scores estimated from different realizations.

Analytic convergence condition in directed network

For the directed network, the process of proof is similar to the undirected network, the difference is that for individual i, only its out-neighbors contribute to f_i, and only its in-neighbors contribute to g_i, similarly, we convert it to the fixed-point problem $x = G(x)$, and get the square of the Jacobian matrix as follows:

$$\mathbf{J^2} = \begin{pmatrix} \mathbf{FAY^{-2}EA^TX^{-2}} & \mathbf{0} \\ \mathbf{0} & \mathbf{EA^TX^{-2}FAY^{-2}} \end{pmatrix} \tag{12}$$

The two blocks $\mathbf{FAY^{-2}EA^TX^{-2}}$ and $\mathbf{EA^TX^{-2}FAY^{-2}}$ with same eigenvalues. $\mathbf{FAY^{-2}EA^TX^{-2}}$ equals to:

$$\begin{pmatrix} \frac{1}{f_1 x_1^2}\sum_j A_{1j}A_{1j}\frac{1}{g_j y_j^2} & \frac{1}{f_1 x_2^2}\sum_j A_{1j}A_{2j}\frac{1}{g_j y_j^2} & \cdots & \frac{1}{f_1 x_N^2}\sum_j A_{1j}A_{Nj}\frac{1}{g_j y_j^2} \\ \frac{1}{f_2 x_1^2}\sum_j A_{2j}A_{1j}\frac{1}{g_j y_j^2} & \frac{1}{f_2 x_2^2}\sum_j A_{2j}A_{2j}\frac{1}{g_j y_j^2} & \cdots & \frac{1}{f_2 x_N^2}\sum_j A_{2j}A_{Nj}\frac{1}{g_j y_j^2} \\ \vdots & \vdots & \ddots & \vdots \\ \frac{1}{f_N x_1^2}\sum_j A_{Nj}A_{1j}\frac{1}{g_j y_j^2} & \frac{1}{f_N x_2^2}\sum_j A_{Nj}A_{2j}\frac{1}{g_j y_j^2} & \cdots & \frac{1}{f_N x_N^2}\sum_j A_{Nj}A_{Nj}\frac{1}{g_j y_j^2} \end{pmatrix} \tag{13}$$

We set x_i^* and y_i^* both equal to I_0 as the initial condition, then the above matrix is simplified as

$$\mathbf{Q} = \begin{pmatrix} \frac{1}{k_1^{out}}\sum_j A_{1j}A_{1j}\frac{1}{k_j^{in}} & \frac{1}{k_1^{out}}\sum_j A_{1j}A_{2j}\frac{1}{k_j^{in}} & \cdots & \frac{1}{k_1^{out}}\sum_j A_{1j}A_{Nj}\frac{1}{k_j^{in}} \\ \frac{1}{k_2^{out}}\sum_j A_{2j}A_{1j}\frac{1}{k_j^{in}} & \frac{1}{k_2^{out}}\sum_j A_{2j}A_{2j}\frac{1}{k_j^{in}} & \cdots & \frac{1}{k_2^{out}}\sum_j A_{2j}A_{Nj}\frac{1}{k_j^{in}} \\ \vdots & \vdots & \ddots & \vdots \\ \frac{1}{k_N^{out}}\sum_j A_{Nj}A_{1j}\frac{1}{k_j^{in}} & \frac{1}{k_N^{out}}\sum_j A_{Nj}A_{2j}\frac{1}{k_j^{in}} & \cdots & \frac{1}{k_N^{out}}\sum_j A_{Nj}A_{Nj}\frac{1}{k_j^{in}} \end{pmatrix} \tag{14}$$

Similarly, we use Gershgorin circle theorem [9] to prove that the modulo of the maximum eigenvalue (spectral radius) of the matrix $\mathbf{Q}$ is small than or equal to 1, which can turn to prove the sum of arbitrary row's element is small than or equal to 1, that is to prove $\frac{1}{k_i^{out}}\sum_j\sum_l A_{ij}A_{lj}\frac{1}{k_j^{in}} \leq 1$ holds. When the out-degree of i is 1 and i's out-neighbor only connects with i, we get $\frac{1}{k_i^{out}}\sum_j\sum_l A_{ij}A_{lj}\frac{1}{k_j^{in}} = 1$ holds, otherwise, it's small than 1. When k_i^{out} fixed and big than 1, to maximum $\sum_j\sum_l A_{ij}A_{lj}\frac{1}{k_j^{in}}$, we get two cases, first is all i's out-neighbors don't connect mutually and with $k_{in} = 1$, in such a case, we get $\sum_j\sum_l A_{ij}A_{lj}\frac{1}{k_j^{in}} = k_i^{out}$, second is all i's out-neighbors are fully connected (We can say its out clustering coefficient $c_i^{out} = 1$), in such a case, $\sum_j\sum_l A_{ij}A_{lj}\frac{1}{k_j^{in}} = k_i^{out}$ as well, except for the above two cases, in any other condition, we get $\sum_j\sum_l A_{ij}A_{lj}\frac{1}{k_j^{in}} < k_i^{out}$, so we proved for matrix $\mathbf{Q}$, the sum of each row's elements small than or equal to 1 when we set x_i^* and y_i^* both equal to nonnegative I_0, according to the Gershgorin circle theorem, we know the spectral radius $\rho(\mathbf{Q}) \leq 1$, finally we proved $\rho(\mathbf{J}) \leq 1$. Besides, the case $\rho(\mathbf{J}) = 1$ if and only if for arbitrary i, its out clustering coefficient c_i^{out} is either 0 or 1 (In this case, sum of each row of matrix $\mathbf{Q}$ is 1, getting $\mathbf{Q}$ with maximum eigenvalue 1, which finally lead to $\rho(\mathbf{J}) = 1$), In other case, we have $\rho(\mathbf{J}) < 1$. One more thing we need to notice is that if there are some individuals' k_{out} is 0, the above process can also hold, what we need to do is to divide the whole directed network into several directed sub-networks and prove their convergence respectively. The above proof is also checked by numerical simulation, we set the initial condition $I^{(0)}$ and $S^{(0)}$ from 0.001 to $10,000$ (same as un-directed network) to iterate the algorithm and find the results keep consistent. Similarly, if we choose different initial conditions (I^0 and S^0 are not the same), in the four large-scale empirical networks, the algorithm also converges to a unique solution.

7. Supplementary Note 6: Numeric results on the convergence of the IS algorithm

For both the small-scale and large-scale datasets, the convergence criteria are the error $\sum_i^N |I_i^{(n+1)} - I_i^{(n)}|/N < 10^{-5}$ and $\sum_i^N |S_i^{(n+1)} - S_i^{(n)}|/N < 10^{-5}$ hold, here N is the size of dataset, and $I_i^{(n)}$ ($S_i^{(n)}$) is the influence (susceptibility) value of individuals i in n-th iteration.

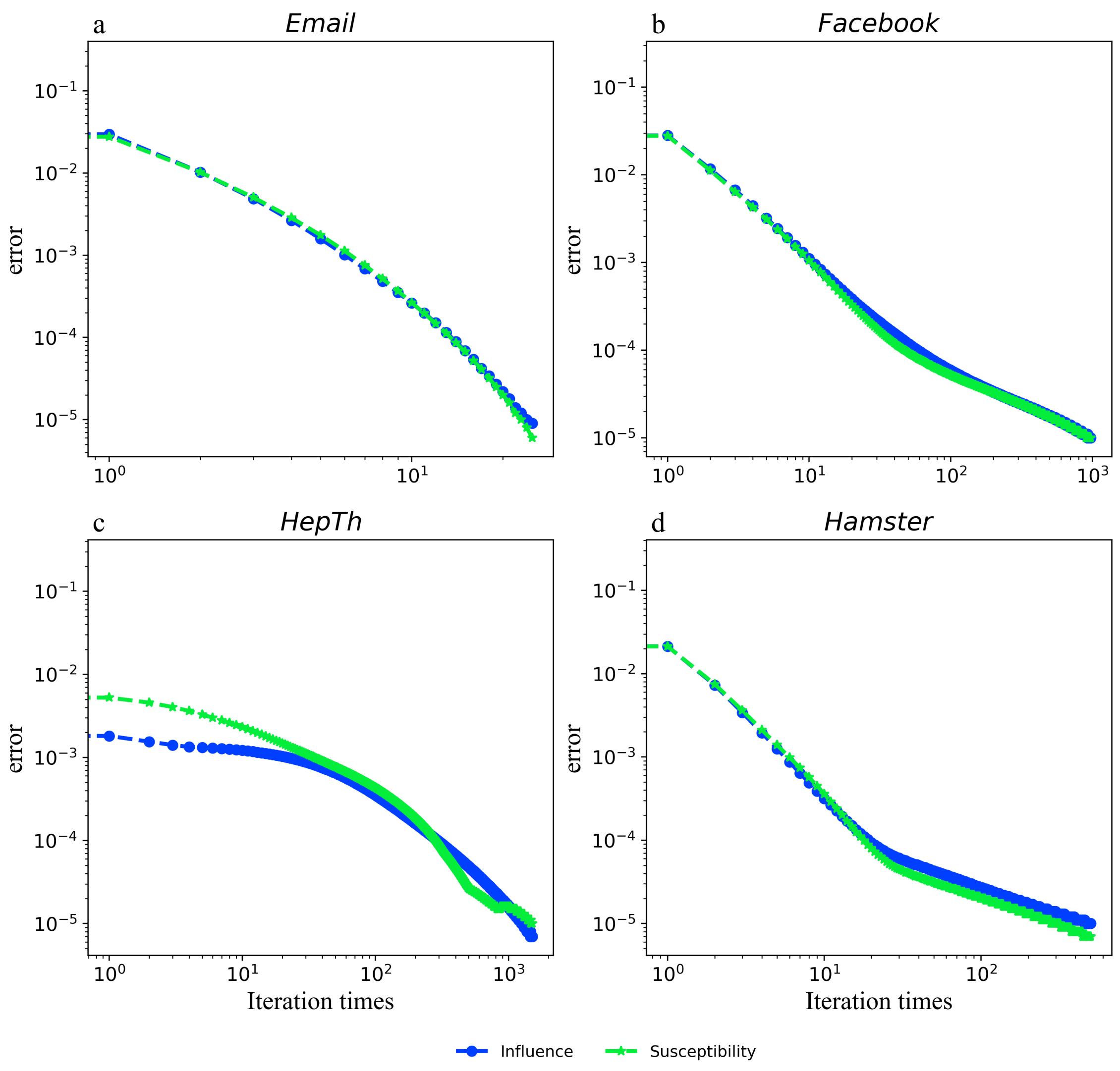

Fig. S9 Convergence plots of the four small-scale datasets. The correlation between iteration times and the error of I (S) of the four small-scale datasets.

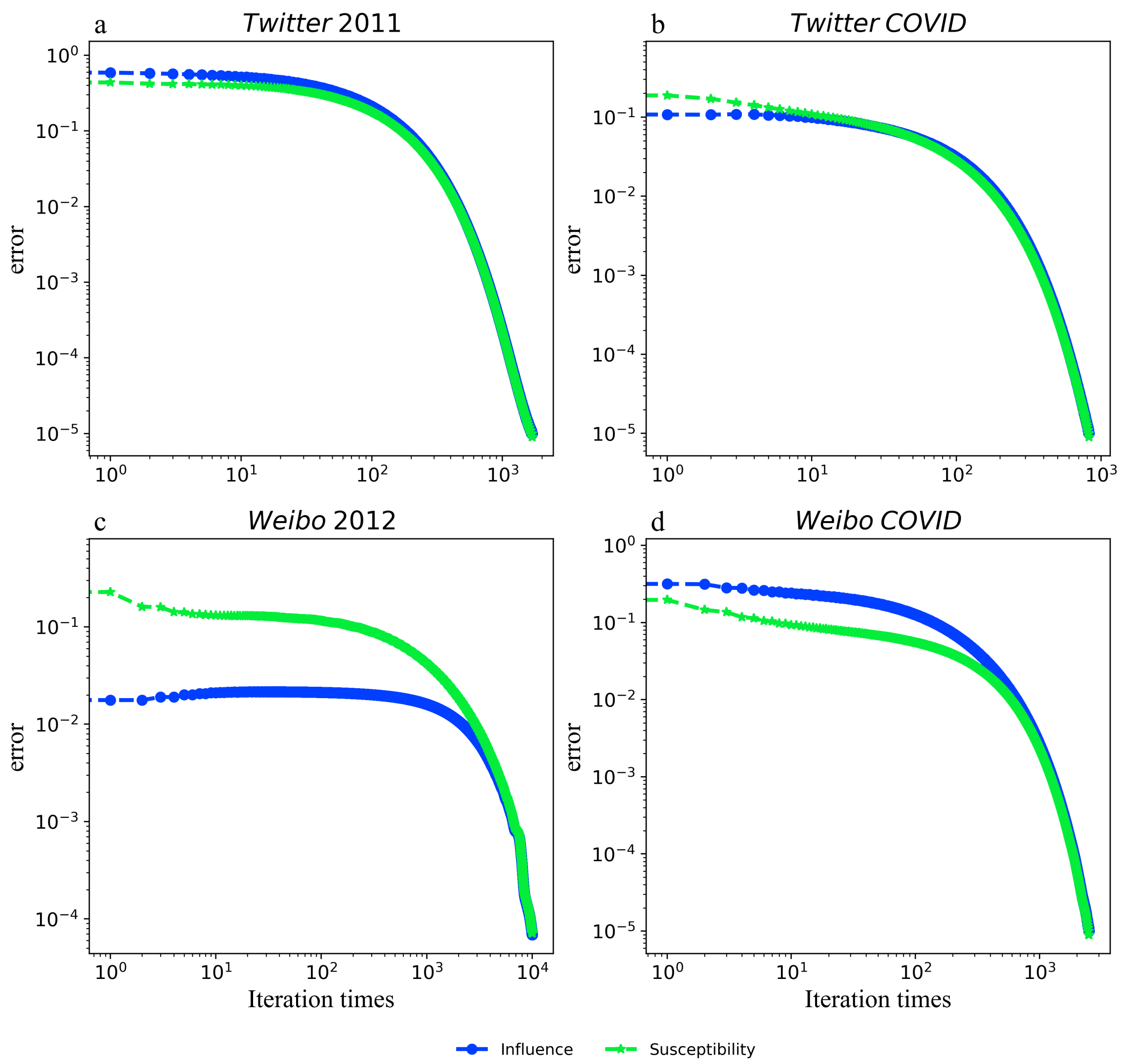

Fig. S10 Convergence plots of the four large-scale datasets. The correlation between iteration times and the error of I (S) of the four large-scale datasets with empirical spreading events.

8. Supplementary Note 7: Linear regression analysis

8.1. Variance inflation factor

We use the variance inflation factor (VIF) to analyze the potential collinearity problem of features. We present the VIF of the 2 empirical datasets as follows. The tables S6-S10 show the VIF scores of 5 sub-datasets of *Weibo COVID*; the tables S11-S15 show the VIF scores of 5 sub-datasets of *Twitter COVID*. For a given table, the first column denotes the feature name; the second column denotes the VIF score that considered all features; the third column denotes the VIF score after removing the features that the VIF higher than 10. We can conclude that the two features *outdegree* and *total susceptibility* are highly correlated.

features	full model	restricted model
outdegree	79.07	-
indegree	2.70	2.70
Pagerank	2.29	2.29
k-core	1.82	1.68
influence	1.25	1.21
susceptibility	1.31	1.31
IS total score	1.83	1.34
total susceptibility	87.64	-
total influence	2.81	1.42

Table S6 The VIF of sub-dataset 1 of *Weibo COVID*

features	full model	restricted model
outdegree	67.86	-
indegree	2.80	2.80
Pagerank	2.37	2.38
k-core	1.88	1.74
influence	1.33	1.29
susceptibility	1.31	1.30
IS total score	2.07	1.20
total susceptibility	80.31	-
total influence	1.90	1.42

Table S7 The VIF of sub-dataset 2 of *Weibo COVID*

features	full model	restricted model
outdegree	58.59	-
indegree	2.98	2.97
Pagerank	2.53	2.53
k-core	1.97	1.83
influence	1.29	1.26
susceptibility	1.27	1.27
IS total score	1.75	1.45
total susceptibility	65.08	-
total influence	2.54	1.38

Table S8 The VIF of sub-dataset 3 of *Weibo COVID*

features	full model	restricted model
outdegree	52.27	-
indegree	2.28	2.27
Pagerank	2.07	2.07
k-core	1.97	1.86
influence	1.21	1.19
susceptibility	1.28	1.28
IS total score	1.64	1.42
total susceptibility	62.10	-
total influence	3.21	1.41

Table S9 The VIF of sub-dataset 4 of *Weibo COVID*

features	full model	restricted model
outdegree	19.20	-
indegree	3.63	3.61
Pagerank	3.07	3.06
k-core	1.92	1.75
influence	1.30	1.27
susceptibility	1.31	1.31
IS total score	1.60	1.33
total susceptibility	22.28	-
total influence	2.11	1.27

Table S10 The VIF of sub-dataset 5 of *Weibo COVID*

features	full model	restricted model
outdegree	128.83	-
indegree	3.53	3.49
Pagerank	1.90	1.90
k-core	3.56	3.44
influence	1.32	1.31
susceptibility	1.65	1.65
IS total score	2.33	1.04
total susceptibility	148.75	-
total influence	7.63	1.23

Table S11 The VIF of sub-dataset 1 of *Twitter COVID*

features	full model	restricted model
outdegree	107.68	-
indegree	3.44	3.40
Pagerank	1.84	1.84
k-core	3.45	3.34
influence	1.31	1.31
susceptibility	1.64	1.64
IS total score	2.11	1.05
total susceptibility	143.95	-
total influence	9.46	1.24

Table S12 The VIF of sub-dataset 2 of *Twitter COVID*

features	full model	restricted model
outdegree	73.90	-
indegree	3.42	3.39
Pagerank	1.82	1.82
k-core	3.40	3.29
influence	1.30	1.30
susceptibility	1.63	1.62
IS total score	2.07	1.03
total susceptibility	97.35	-
total influence	10.13	1.22

Table S13 The VIF of sub-dataset 3 of *Twitter COVID*

features	full model	restricted model
outdegree	110.42	-
indegree	3.44	3.40
Pagerank	1.83	1.83
k-core	3.42	3.30
influence	1.31	1.31
susceptibility	1.65	1.64
IS total score	2.20	1.04
total susceptibility	131.79	-
total influence	10.14	1.23

Table S14 The VIF of sub-dataset 4 of *Twitter COVID*

features	full model	restricted model
outdegree	105.83	-
indegree	3.26	3.22
Pagerank	1.72	1.71
k-core	3.33	3.22
influence	1.30	1.30
susceptibility	1.59	1.59
IS total score	2.51	1.04
total susceptibility	132.68	-
total influence	9.47	1.22

Table S15 The VIF of sub-dataset 5 of *Twitter COVID*

8.2. Linear mixed effect model

The linear mixed effect model is a regression model that incorporates both fixed effects and random effects [10]. It is used to analyze samples that have a hierarchical or nested structure, where samples are grouped within higher-level units. In a linear mixed effects model, fixed effects represent the population-level effects that are assumed to be constant across all groups or clusters. On the other hand, random effects represent the variation between the different groups or clusters in the data. Random effects are treated as random variables drawn from a certain distribution, and they capture the variations at the group level.

Secondly, we consider the linear mixed effect model to analyze the contribution of different features and fix the random effect caused by different groups (sub-datasets). Specifically, we use the `smf.mixedlm` function in Python `statusmodel` package to implement the linear mixed effect model, except the 7 features (excluded *outdegree* and *total susceptibility*) considered, we also add the sub-dataset's group as a feature, that is for the individuals in sub-dataset 1, we denote its group id as 1. Then we combine all the individuals' 8 features (independent variables) and spreading capacity (dependent variable) in 5 sub-datasets together, inputting them into the linear mixed effect model and setting the group as a random effect. Finally, we get the following two tables S16, S17 for *Weibo COVID* and *Twitter COVID*, respectively.

From the tables, we find that in the two datasets, the IS-based features (*IS total score*) contribute more positive weights compared to structure-based features. Importantly, we find that the IS total score, the total influence, and the k-core centrality [6] are the only variables with a significant association with the users' spreading success in both Twitter and Weibo COVID. Since the *Group Var* coefficient is very small for both two datasets, it implies that there is less variation among different groups. This indicates that the random effect between groups has less impact on the dependent variable, while the fixed effects are more crucial.

Table S16 Mixed Linear Model Regression Results of *Weibo COVID*. The interpretation of the parameters is as follows: 'Coef' denotes the coefficient of the features; 'Std Err' represents the standard error of the coefficient; 'z' is computed by dividing the coefficient estimate by its standard error; 'P> |z|' represents the probability (p-value) of observing a z-value as extreme as the one calculated, assuming the null hypothesis that the coefficient is equal to zero; '[0.025 0.975]' is the confidence interval of the estimated coefficient.

Model:	MixedLM	Dependent Variable:	spreading capacity
No. Observations:	163746	Method:	REML
No. Groups:	5	Scale:	571.4043
Min. group size:	29984	Log-Likelihood:	-752096.8908
Max. group size:	34553	Converged:	Yes
Mean group size:	32749.2		

	Coef.	Std.Err.	z	P> |z|	[0.025	0.975]
Intercept	3.017	0.134	22.544	0.000	2.755	3.279
indegree	-0.107	0.098	-1.092	0.275	-0.300	0.085
PageRank	0.084	0.091	0.920	0.358	-0.095	0.263
k-core	0.306	0.077	3.964	0.000	0.155	0.457
influence	-0.034	0.068	-0.498	0.619	-0.168	0.100
susceptibility	-0.116	0.068	-1.697	0.090	-0.249	0.018
IS total score	3.125	0.065	47.943	0.000	2.997	3.252
total influence	0.874	0.068	12.837	0.000	0.741	1.008
Group Var	0.072	0.003				

Table S17 Mixed Linear Model Regression Results of *Twitter COVID*. The interpretation of the parameters is as follows: 'Coef' denotes the coefficient of the features; 'Std Err' represents the standard error of the coefficient; 'z' is computed by dividing the coefficient estimate by its standard error; 'P> |z|' represents the probability (p-value) of observing a z-value as extreme as the one calculated, assuming the null hypothesis that the coefficient is equal to zero; '[0.025 0.975]' is the confidence interval of the estimated coefficient.

Model:	MixedLM	Dependent Variable:	spreading capacity
No. Observations:	1889757	Method:	REML
No. Groups:	5	Scale:	3216.7709
Min. group size:	364286	Log-Likelihood:	-10312429.4535
Max. group size:	386275	Converged:	Yes
Mean group size:	377951.4		

	Coef.	Std.Err.	z	P> |z|	[0.025	0.975]
Intercept	4.388	0.072	60.635	0.000	4.246	4.530
indegree	-2.332	0.070	-33.424	0.000	-2.469	-2.195
PageRank	0.169	0.053	3.184	0.001	0.065	0.272
k-core	3.077	0.063	49.171	0.000	2.954	3.199
influence	0.451	0.043	10.586	0.000	0.368	0.535
susceptibility	-0.271	0.044	-6.203	0.000	-0.357	-0.185
IS total score	5.548	0.042	132.082	0.000	5.466	5.630
total influence	2.279	0.046	50.048	0.000	2.190	2.368
Group Var	0.018	0.000				

9. Supplementary Note 8: Classification algorithms

We use three classification algorithms to predict superspreaders (binary classification), including Random Forest[11], XGBoost[12], and multiplayer perceptrons[13]. Here, the superspreaders are defined as the individuals with top spreading ability, where a user's spreading ability is defined as the average size of the cascades initiated by the user. We adopt Python's `sklearn` package (version number: 0.24.1) to deploy the three algorithms. For the three algorithms, we use the `sklearn` GridSearchCV function to optimize some of the algorithm's hyper-parameters. Following are the details of the algorithm's parameters setting.

Random forest algorithm

For the Random forest algorithm [11], there are four important hyper-parameters: max depth of a tree (max_depth); minimum samples required for a node to split ($min\_samples\_split$); minimum samples of a leaf node ($min\_samples\_leaf$); and the number of trees in the random forest algorithm ($n\_estimators$). In our experiments, to get stable prediction results, we fix the $n\_estimators = 200$ for all the models. We use the Python `sklearn GridSearchCV` function to determine the remaining best performance hyper-parameters and the range of the hyper-parameters are max depth (max_depth): 5, 10, 15, 20, 30, 40; minimum samples for split ($min\_samples\_split$): 20, 40, 60, 80; minimum samples of leaf node ($min\_samples\_leaf$): 20, 30, 40, 50. Specifically, for a given random forest model, e.g., the structure-based (Diffusion) model. In the training set, we use the `GridSearchCV` function to determine the best performance hyper-parameters. Other models are similar. Finally, for *Weibo COVID*, a total of 4 groups of best performance hyper-parameters are chosen; for *Twitter COVID*, a total of 6 groups of best performance hyper-parameters are chosen.

The Random Forest algorithm is the ensemble of decision trees. Given independent variables $x_i \in R^n$ and the dependent variables $y \in R$, a decision tree recursively partitions the feature space such that the samples with the same labels or similar target values are grouped together. Let the data at node m be represented by Q_m with n_m samples. For each candidate split $\theta = (j, t_m)$ consisting of a feature j and threshold t_m, partition the data into $Q_m^{left}(\theta)$ and $Q_m^{right}(\theta)$ subsets

$$Q_m^{left}(\theta) = (x, y)|x_j \le t_m, Q_m^{right}(\theta) = Q_m \backslash Q_m^{left}(\theta). \tag{15}$$

The quality of a candidate split of node m is then computed using the entropy function $H()$ (defined later) as the loss function.

$$G(Q_m, \theta) = \frac{n_m^{left}}{n_m} H(Q_m^{left}(\theta)) + \frac{n_m^{right}}{n_m} H(Q_m^{right}(\theta)) \tag{16}$$

Then, select the parameters that minimize the entropy

$$\theta^* = argmin_\theta G(Q_m, \theta). \tag{17}$$

Finally, recurse for subsets $Q_m^{left}(\theta)$ and$Q_m^{right}(\theta)$ until the maximum allowable depth is reached, $n_m < min_{samples}$ or $n_m = 1$.

We use the entropy function as the loss function. For classification outcome taking on values k (k = 0, 1), for node m, let $p_{mk} = \frac{1}{n_m} \sum_{y \in Q_m} I(y = k)$ be the proportion of class k observations in the node m. If m is a terminal node, *predict_proba* for this region is set to p_{mk}. The entropy loss function is defined as follows.

$$H(Q_m) = -\sum^{k} p_{mk} log(p_{mk}). \tag{18}$$

The corresponding details of the above classification criterion and loss function refer to the technical document of `sklearn` package `https://scikit-learn.org/stable/modules/tree.html#mathematical-formulation`.

XGBoost

XGBoost is a newly proposed gradient-boosting algorithm that can be used for classification tasks [12]. It is a powerful ensemble learning method that combines the predictions of multiple weak learner models to create a strong learner model. In our experiment, we use the Python `xgboost` package to implement the XGBoost algorithm. Similar to the random forest algorithm. We fix the $n\_estimators = 200$ for all the models. Then, we use the Python `sklearn GridSearchCV` function to determine the remaining best-performance hyper-parameters. The range of the hyper-parameters is max depth (max_depth): 5, 10, 20, 30, 40; learning rate ($learning\_rate$): 0.001, 0.01, 0.1; regularization parameter (reg_alpha): 0.001, 0.01, 0.1, 1. For *Weibo COVID*, total 4 groups of best performance hyper-parameters are chosen; for *Twitter COVID*, total 6 groups of best performance hyper-parameters are chosen.

Multilayer Perceptron

A Multilayer Perceptron (MLP) [13] is a type of artificial neural network that consists of multiple layers of interconnected nodes, called neurons. It is a feedforward neural network where information flows in one direction only, from the input layer through the hidden layers to the output layer. Similar to the above two algorithms. In the experiments, we use the Python `sklearn` package to implement the MLP algorithm and the `GridSearchCV` function to determine the best performance hyper-parameters. The hyper-parameters space are hidden layer size ($hidden\_layer\_sizes$): (100, 100), (200, 200), (300, 300); max iteration (max_iter):1000, 2000; regularization parameter ($alpha$): 0.00001, 0.0001, 0.001, 0.01. For *Weibo COVID*, total 4 groups of best performance hyper-parameters are chosen; for *Twitter COVID*, total 5 groups of best performance hyper-parameters are chosen.

10. Supplementary Note 9: Data collection, sampling, and partition

Data collection

We use four large-scale datasets in the experiments: (i) *Twitter 2011*, (ii) *Twitter COVID*, (iii) *Weibo 2012* and (iv) *Weibo COVID*. The *Twitter 2011* [6] contains $996,525$ spreading events among $901,949$ users from January 23, 2011 to February 8, 2011. The *Twitter COVID* dataset is obtained from[14], which provides the IDs of COVID-related tweets [`https://github.com/thepanacealab/covid19_twitter`]. Using the IDs of these tweets, we crawl the related retweeting cascades from March 22, 2020, to March 29, 2020, obtaining $72,290,030$ spreading events among $12,599,426$ users. Weibo is a Twitter-like social application in China. The *Weibo 2012* dataset includes $31,878,405$ spreading events among $1,340,789$ users from August 30, 2009 to December 26, 2012; it can be downloaded from [`https://www.aminer.cn/influencelocality`]. The *Weibo COVID* dataset collects $24,309,214$ COVID-related spreading events among $2,441,165$ users from December 1, 2019 to March 20, 2020. All the cleaned datasets can be downloaded from [`https://github.com/zervel3/large-scale-social-datasets`].

The users' indegree and outdegree in the four datasets exhibit heavy-tailed distributions (see Figs. S16-S19), which aligns with the heavy tails of online popularity and activity found in previous studies[15]. We note that differently than the indegree and outdegree, the reconstructed influence and susceptibility scores do not follow heavy-tailed distributions, but rather bell-shaped ones (see Figs. S20-S27 for details).

Data sampling

Among the 4 large-scale datasets, *Weibo 2012*, *Twitter 2011*, *Twitter COVID* are publicly available, whereas the *Weibo COVID* dataset is obtained thanks to a collaboration with the Weibo platform. We detail below the sampling procedure for each of the four datasets:

- *Weibo 2012*. To begin with, 100 random users were selected as seed users, and then their followers' and followers' followers were collected. The crawling process produced in total of 1.7 million users and 0.4 billion following relationships among them, with an average of 200 followers per user. For each user, the crawler collected her 1,000 most recent micro-blogs (including tweets and retweets).
- *Twitter 2011*. This dataset is provided by ref. [6]. We refer to the original paper for the sampling procedure used to construct the dataset.
- *Twitter COVID* and *Weibo COVID*. All the tweets (micro-blogs) that contain keywords correlated with COVID-19 are collected. Specifically, the crawler bots crawl the tweets if the tweet includes a keyword among the following ones: #COVID, #COVID19, #VIRUS, #PANDEMIC. All the tweets /messages on Weibo and Twitter that contained at least one among these four keywords were collected within a given period (from December 1, 2019, to March 20, 2020, in *Weibo COVID*; from March 22, 2020, to March 29, 2020, in *Twitter COVID*).

Data partition

For the *Weibo COVID* and *Twitter COVID* datasets, we divide the whole datasets into 6 consecutive non-overlapping windows according to the resharing events' times. We train the Random Forest, XGBoost, and MLP models using individuals that are active both in windows 1 and 2. Specifically, we select individuals that are active in window 1 and are seed nodes in window 2. Then, we use individuals' features in window 1 as the independent variables, and use individuals' average spreading capacity in window 2 as the dependent variable. Note that by only training the algorithms using data from windows 1 and 2, we ensure that all the predictive results from subsequent pairs of windows are out of sample. In each realization of the algorithm, we select 50% of the individuals to train the model. In pairs $(t, t+1)$ of subsequent windows, we test the performance of the trained models by selecting at random 50% of the individuals from window t to predict whether the selected individuals are superspreaders in

window $t+1$. The above process is repeated 50 times. Results in Fig. S30 refer to averages over all the realizations of this process; error bars indicate the standard error of the mean.

11. Supplementary Note 10: IS algorithm discussion and extension

The structure of equations (3) and (4) in the main text resembles that of the equations of the fitness-complexity algorithm for bipartite networks[16, 17], yet our algorithm is designed to apply to unipartite weighted networks. The only information the algorithm requires is historical resharing data, without the need for knowing the detailed follower-followee structure.

We note that through the proposed reconstruction algorithm, we do not impose the constraint that influence and susceptibility scores are within the $(0,1)$ range, which in rare cases could lead to $A_{ij}\,I_i\,S_j > 1$ (e.g., 3.9% in *Weibo 2012*; 3.7% in *Weibo COVID*; 0.0% in *Twitter 2011*; 2.3% in *Twitter COVID*). The reconstructed scores can indeed be interpreted as the solution of a constrained optimization problem[17, 18] where the quadratic form $E(\boldsymbol{I},\boldsymbol{S}) = \sum_{i,j} A_{ij}\,I_i\,S_j = \boldsymbol{I}^T\,\mathsf{A}\,\boldsymbol{S}$ is optimized under the constraints $\prod_i I_i^{f_i} = \prod_i S_i^{g_i} = 1$, without additional constraints on $\boldsymbol{I}$ and $\boldsymbol{S}$. Reconstructed score values larger than one do not pose a problem for analyses where the focus is on the scores' assortativity properties, correlations, and predictive power – e.g., in the "Predicting superspreaders" section, where the scores and their combinations enter as features of the Random Forest algorithm (or alternative XGBoost and MLP algorithms). On the other hand, if one wishes to implement dynamic simulations where the contagion probability is determined by $A_{ij}\,I_i\,S_j$, one would need to ensure that the contagion probabilities are smaller than one. This could be achieved either by rescaling *post-hoc* the reconstructed scores (or the reconstructed contagion probabilities) in such a way that they lie in $[0,1]$, or by adding the additional constraint that the scores' sum is fixed in the optimization problem; we leave these directions to future research.

Note that in principle, the algorithm derived below could be generalized in various ways; for example, to scenarios where the nodes influence and susceptibility scores are not scalars, but p-dimensional vectors[5]; to include j's intrinsic preferences for the content α being reshared; In our model, we followed Aral and Dhillon's assumption, which is that the transmission probability is purely determined by individual's influence and susceptibility scores. Characteristics of the post can be included in the model as well. For example, one could assume the j's decision to reshare is not only influenced by her susceptibility to social influence but also by how much she likes the specific content α being shared. In formulas, one could assume that the probability to reshare a piece of content α is: $p_{ij\alpha} = A_{ij}\,I_i\,S_j\,x_{j\alpha}$, where $xj\alpha$ would capture the intrinsic attractiveness of message α for user j. This would lead to modified equations with extra factors which include j's intrinsic preferences:

$$\begin{aligned} I_i &= \frac{f_i}{\sum_j A_{ij}\,S_j\,X_j}, \\ S_j &= \frac{g_j}{X_j\sum_i A_{ij}\,I_i}, \end{aligned} \tag{19}$$

where $X_j = C^{-1}\sum_\alpha x_{j\alpha}$ represents the j's average preference for the content reshared in the network, and C the total number of reshared pieces of information. The parameter estimation for this model would be more complex, as it would require the estimation of individual's preferences for specific pieces of content, and we leave it for future research.

12. Supplementary Figures

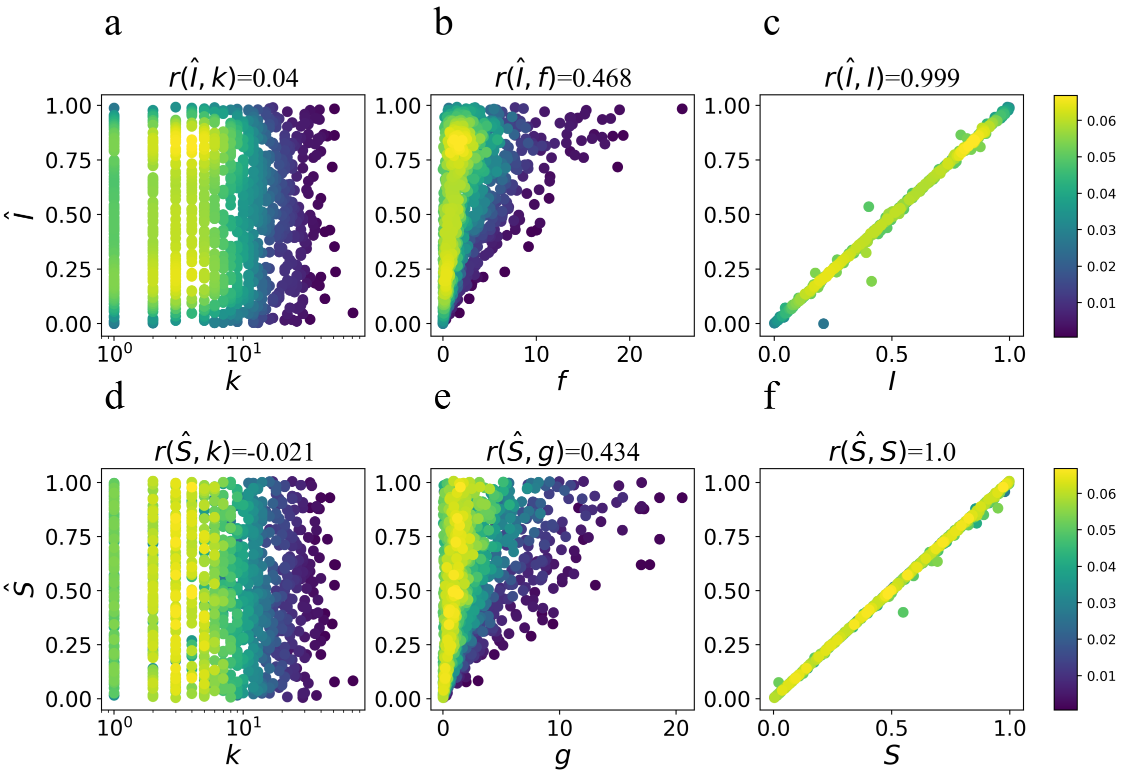

Fig. S11 Reconstructing individuals' ground-truth influence and susceptibility from synthetic spreading events on *Email* network. I, f, S, g, k are individuals' ground-truth influence, outgoing contagion rate, susceptibility, incoming contagion rate, and degree, respectively. $\hat{I}$ and $\hat{S}$ are the reconstructed influence and susceptibility, respectively. $r(\hat{I}, I)$ is the Pearson's coefficient between $\hat{I}$ and I, others are similar. The value of the color bar denotes the normalized probability density (normalized by the maximum probability density of each row) estimated by the Gaussian kernel method [19].

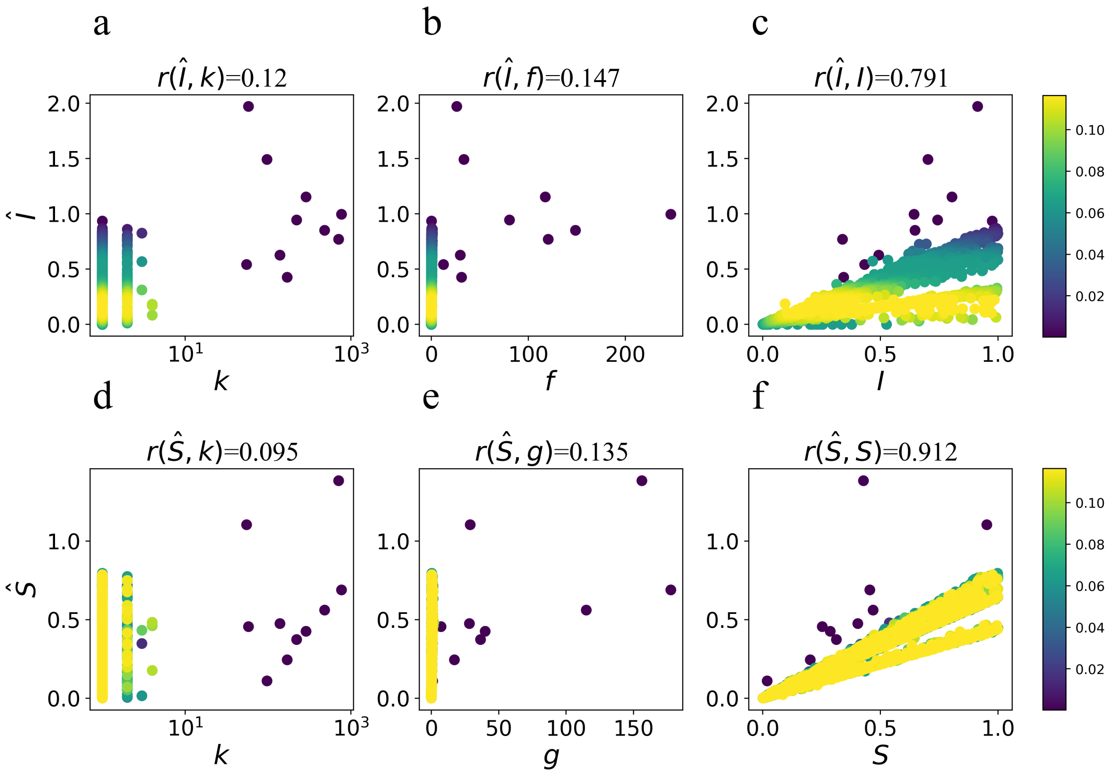

Fig. S12 Reconstructing individuals' ground-truth influence and susceptibility from synthetic spreading events on *Facebook* network. I, f, S, g, and k are individuals' ground-truth influence, outgoing contagion rate, susceptibility, incoming contagion rate, and degree, respectively. $\hat{I}$ and $\hat{S}$ are the reconstructed influence and susceptibility, respectively. $r(\hat{I}, I)$ is the Pearson's coefficient between $\hat{I}$ and I, others are similar.

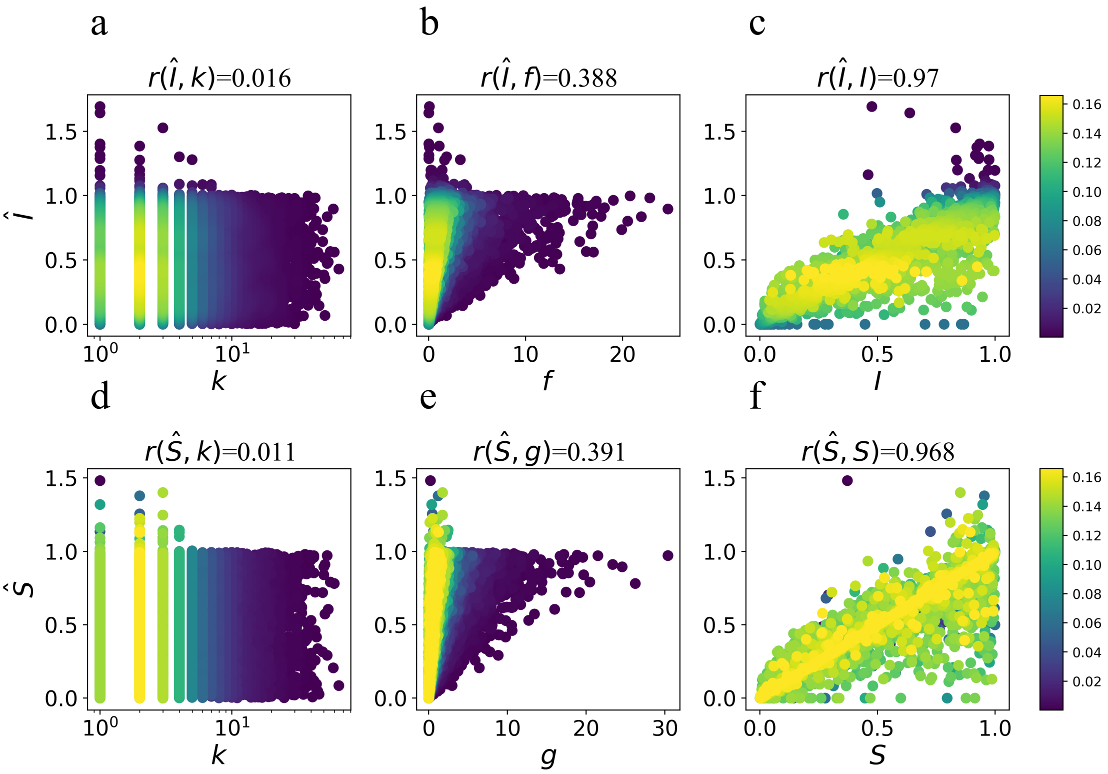

Fig. S13 Reconstructing individuals' ground-truth influence and susceptibility from synthetic spreading events on *HepTh* network. I, f, S, g, and k are individuals' ground-truth influence, outgoing contagion rate, susceptibility, incoming contagion rate, and degree, respectively. $\hat{I}$ and $\hat{S}$ are the reconstructed influence and susceptibility, respectively. $r(\hat{I}, I)$ is the Pearson's coefficient between $\hat{I}$ and I, others are similar.

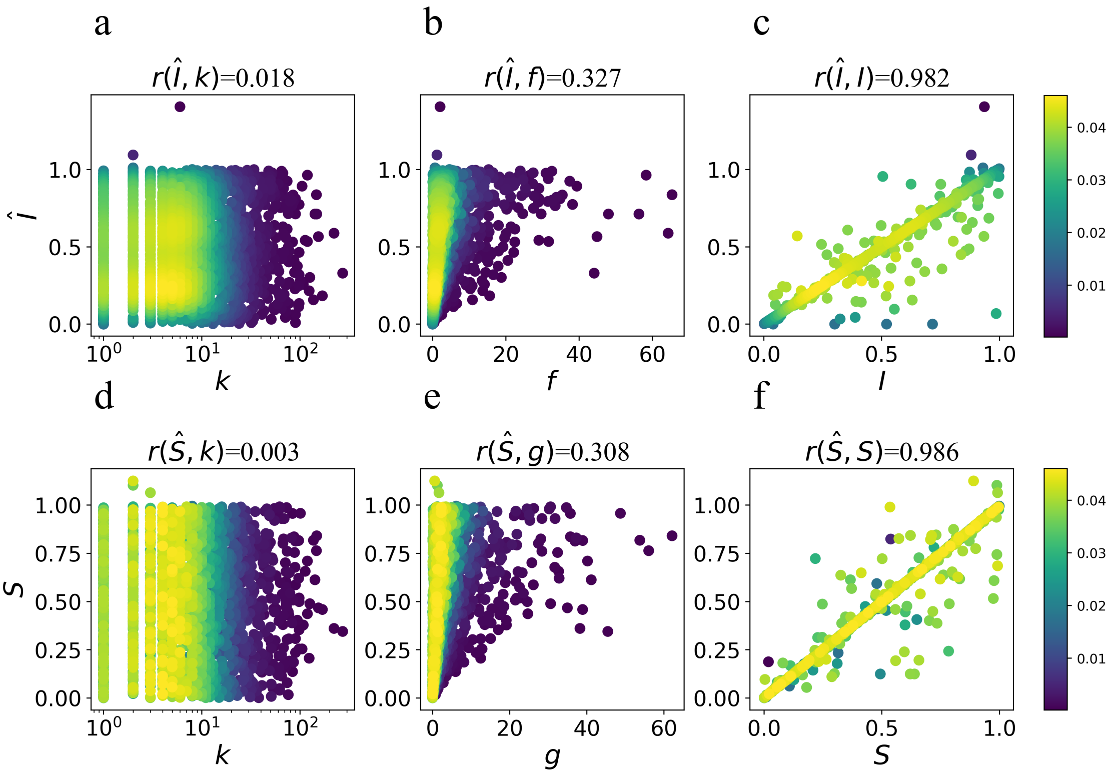

Fig. S14 Reconstructing individuals' ground-truth influence and susceptibility from synthetic spreading events on *Hamster* network. I, f, S, g, and k are individuals' ground-truth influence, outgoing contagion rate, susceptibility, incoming contagion rate, and degree, respectively. $\hat{I}$ and $\hat{S}$ are the reconstructed influence and susceptibility, respectively. $r(\hat{I}, I)$ is the Pearson's coefficient between $\hat{I}$ and I, others are similar.

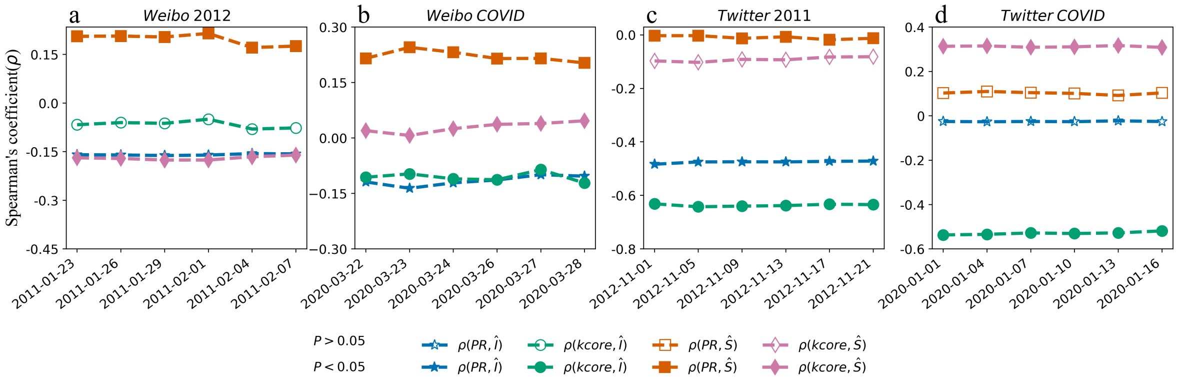

Fig. S15 Empirical correlations between individual-level properties. We divide the spreading events into 6 consecutive non-overlapping periods. The date on the x-axis represents the starting date of each period. In each period, we reconstruct individual influence and susceptibility via the IS algorithm and measure the Spearman's correlation coefficients, ρ, between PageRank (PR), k-core (kcore), influence ($\hat{I}$) and susceptibility ($\hat{S}$). Filled markers denote correlation values that are significantly larger or smaller than the correlation values calculated on randomized networks ($P < 0.05$, see Note 4 for details); empty markers denote correlation values that do not significantly differ from the correlation values calculated on randomized networks ($P > 0.05$).

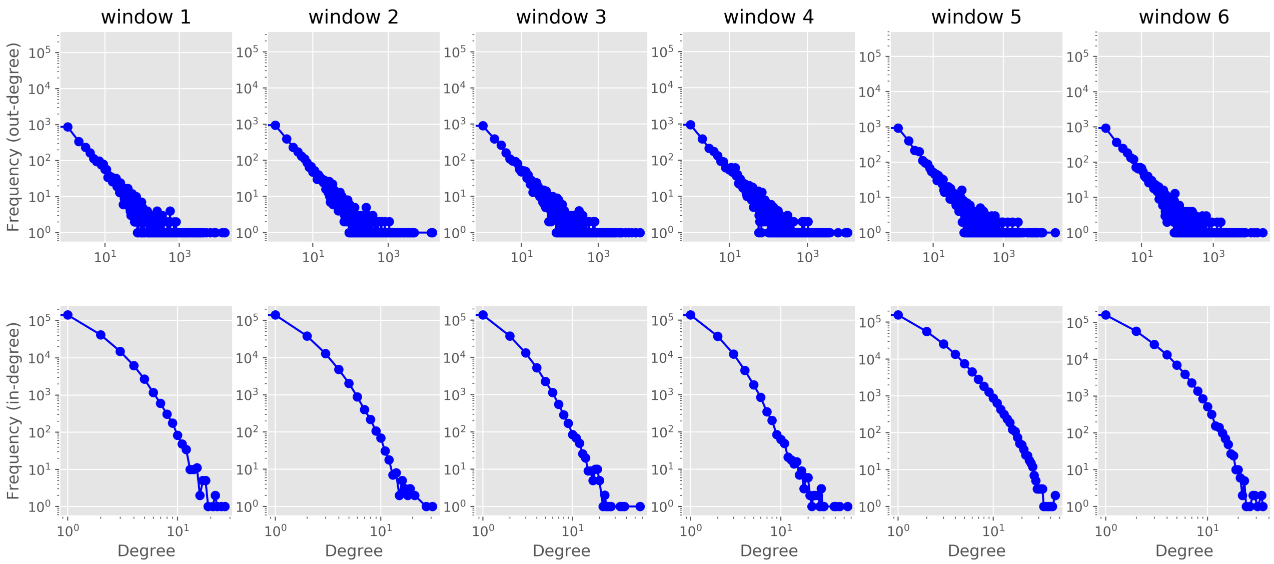

Fig. S16 The degree distribution in the 6 windows of the *Weibo 2012* dataset.

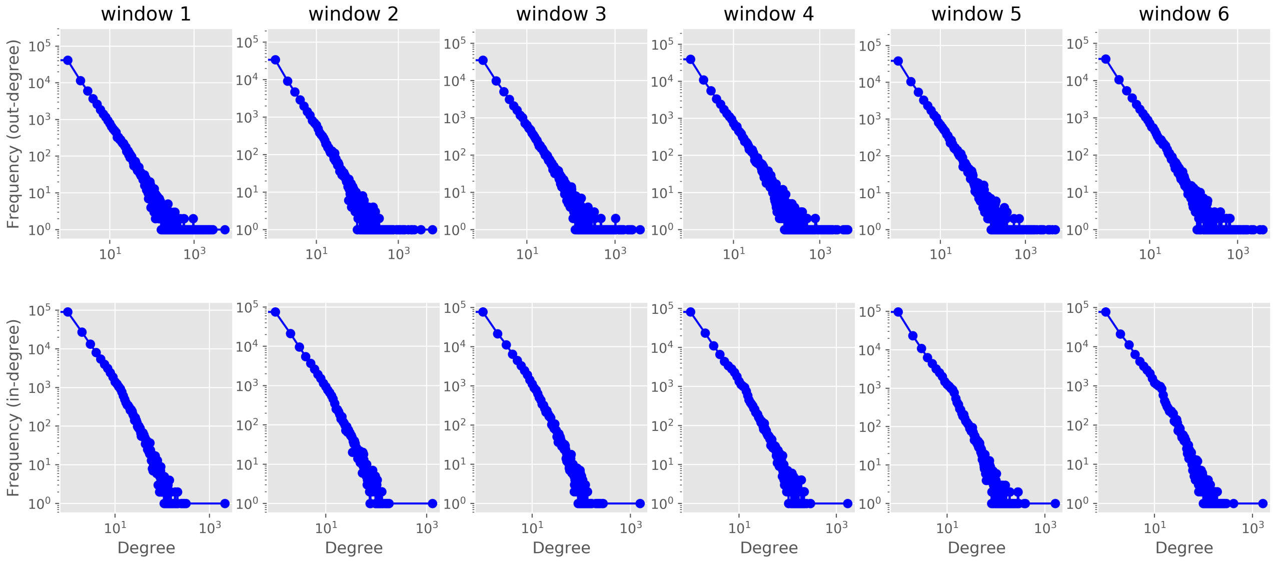

Fig. S17 The degree distribution in the 6 windows of the *Weibo COVID* dataset.

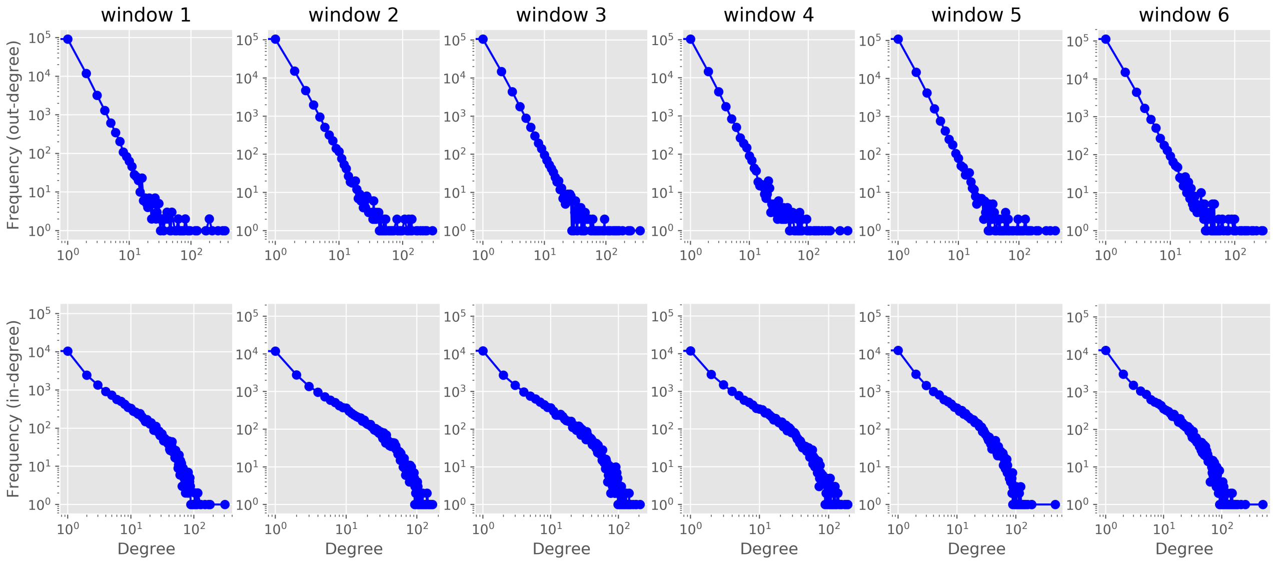

Fig. S18 The degree distribution in the 6 windows of the *Twitter 2011* dataset.

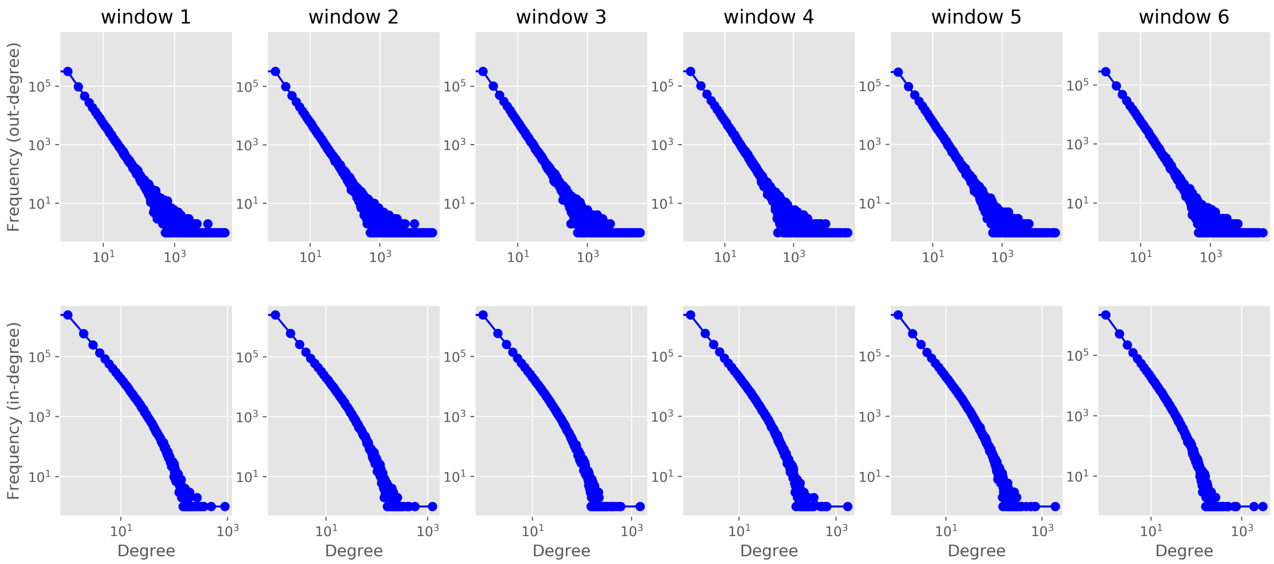

Fig. S19 The degree distribution in the 6 windows of the *Twitter COVID* dataset.

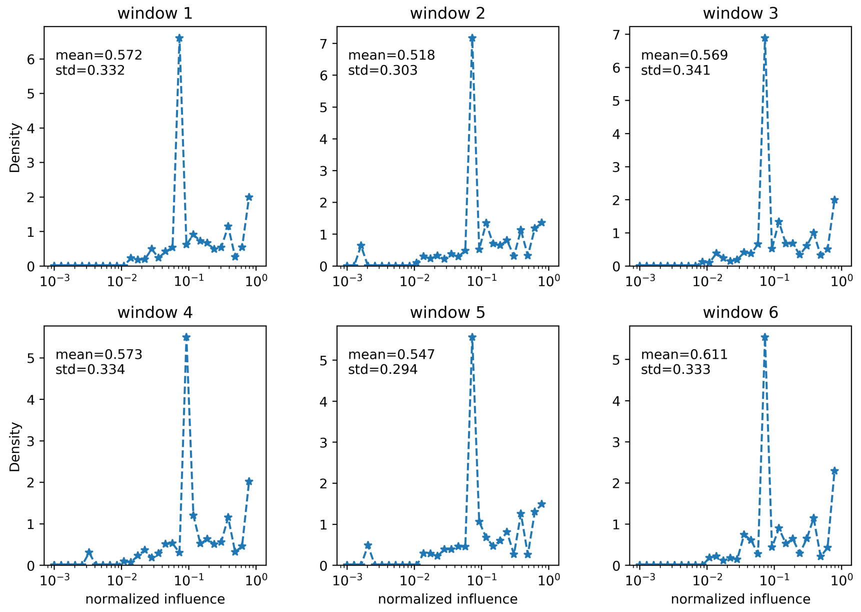

Fig. S20 The influence distribution in the 6 windows of the *Weibo 2012* dataset.

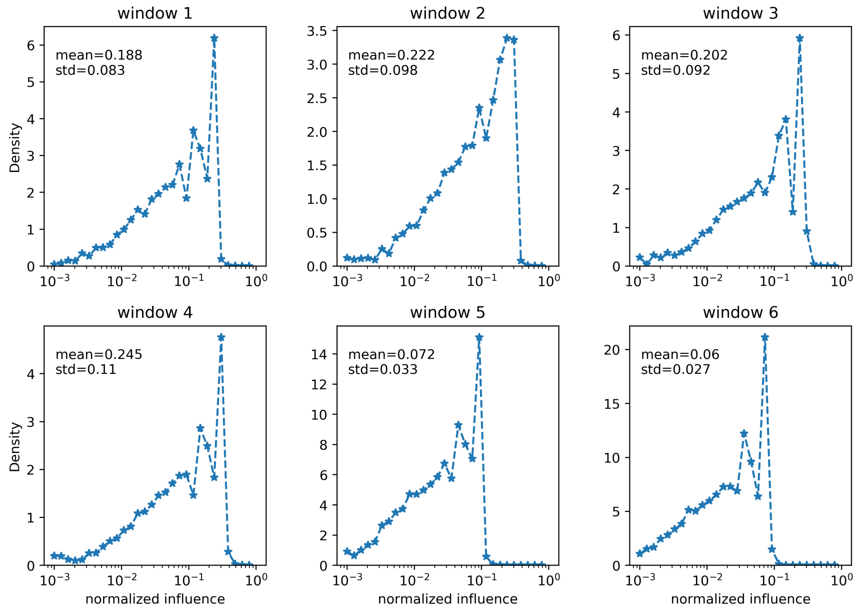

Fig. S21 The influence distribution in the 6 windows of the *Weibo COVID* dataset.

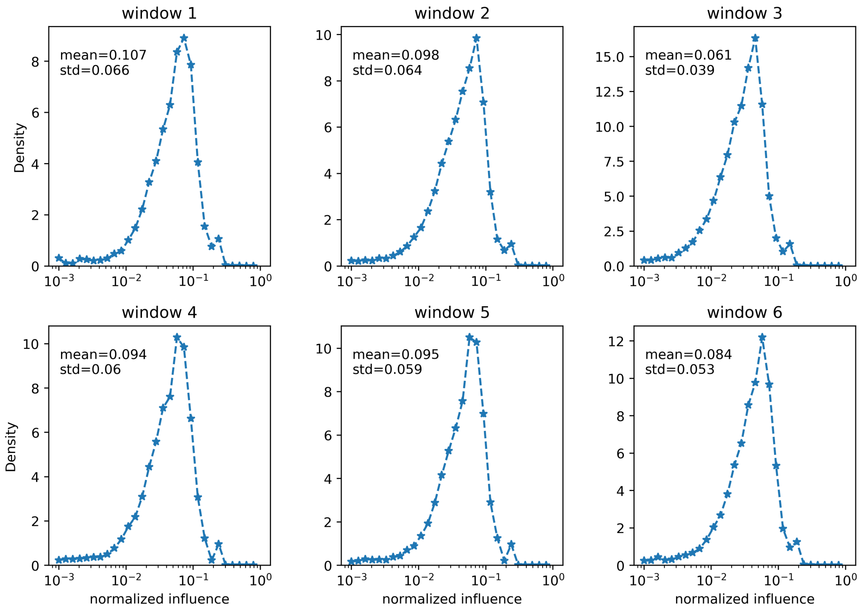

Fig. S22 The influence distribution in the 6 windows of the *Twitter 2011* dataset.

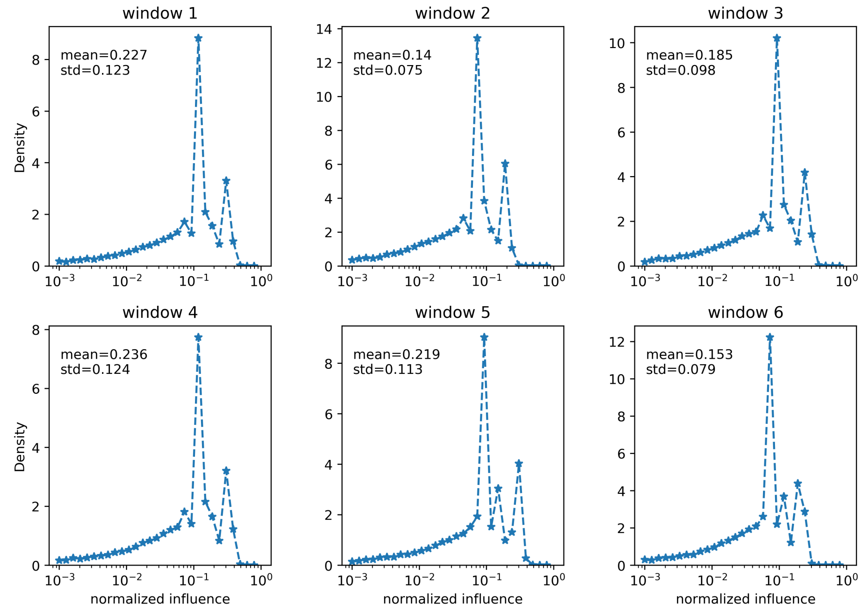

Fig. S23 The influence distribution in the 6 windows of the *Twitter COVID* dataset.

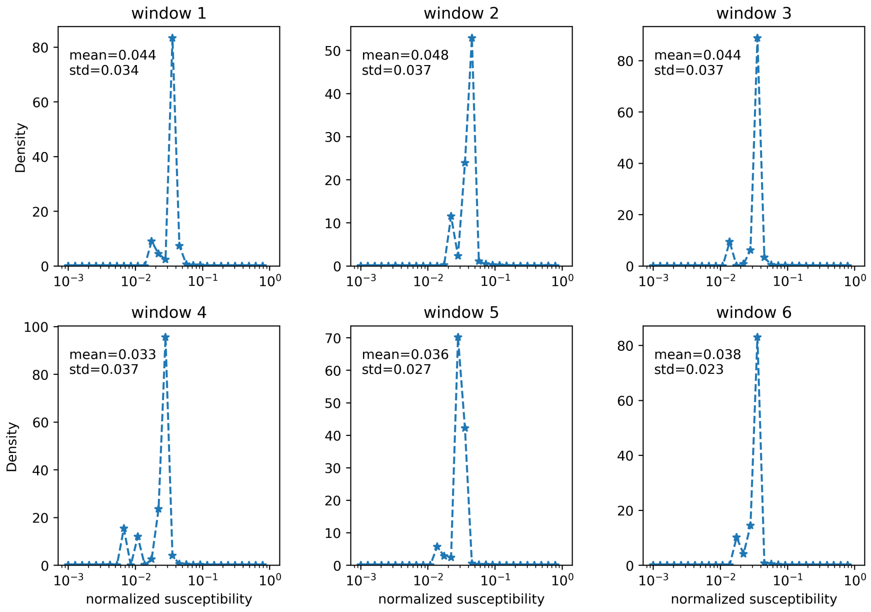

Fig. S24   The susceptibility distribution in the 6 windows of the *Weibo 2012* dataset.

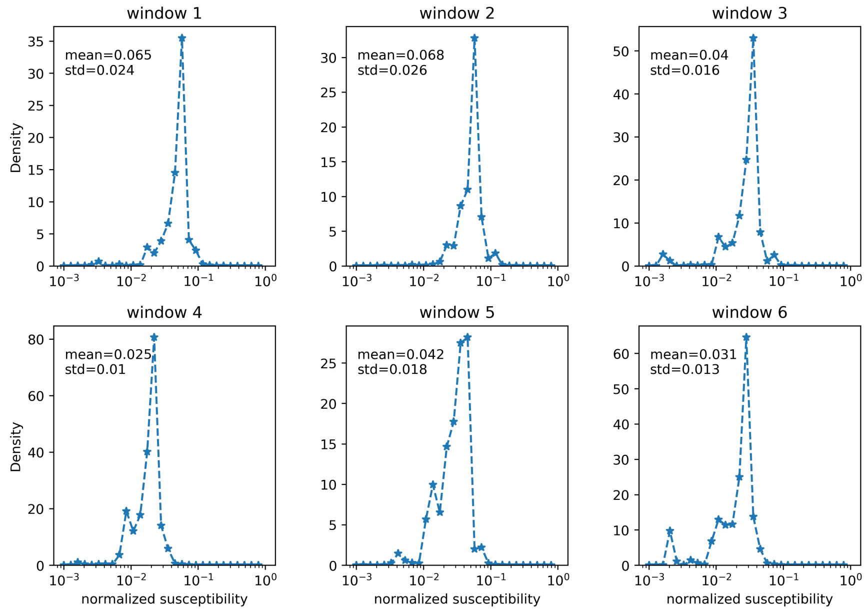

Fig. S25   The susceptibility distribution in the 6 windows of the *Weibo COVID* dataset.

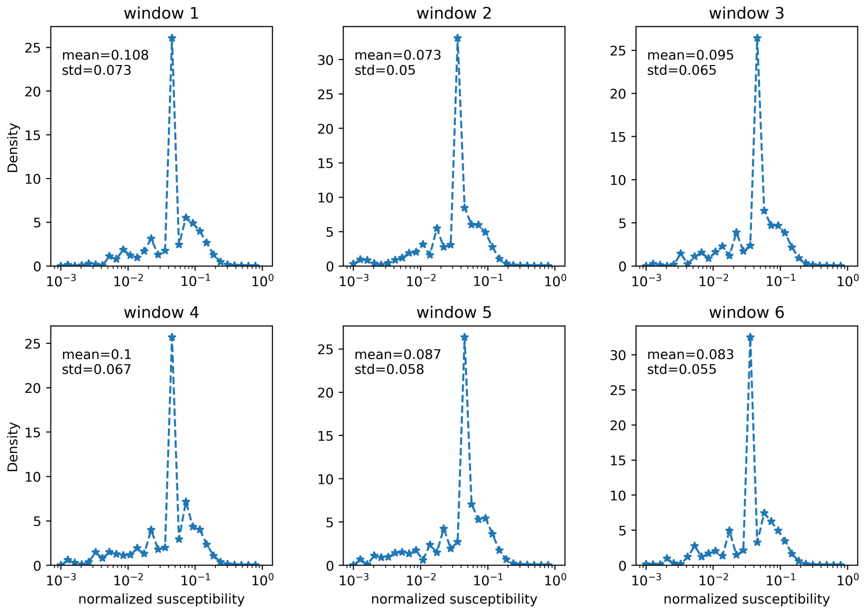

Fig. S26 The susceptibility distribution in the 6 windows of the *Twitter 2011* dataset.

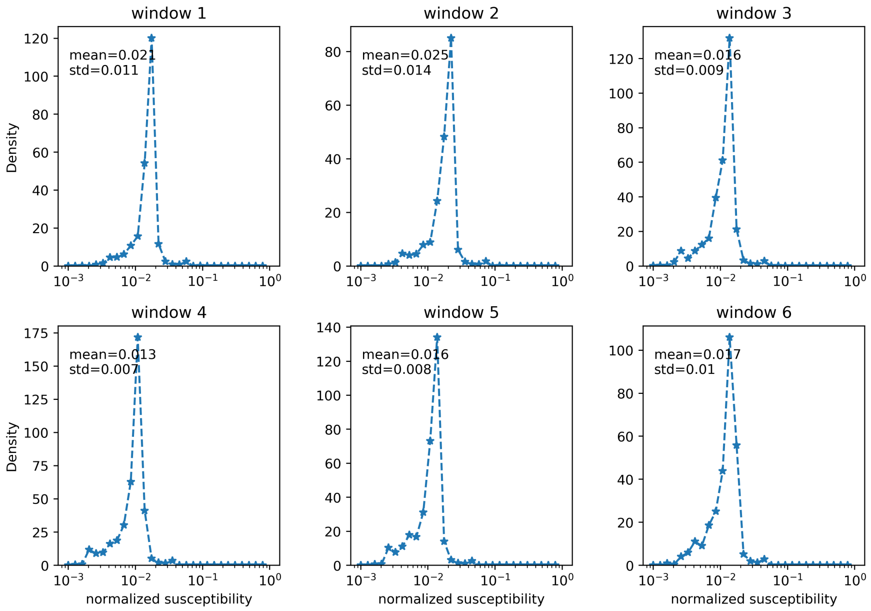

Fig. S27 The susceptibility distribution in the 6 windows of the *Twitter COVID* dataset.

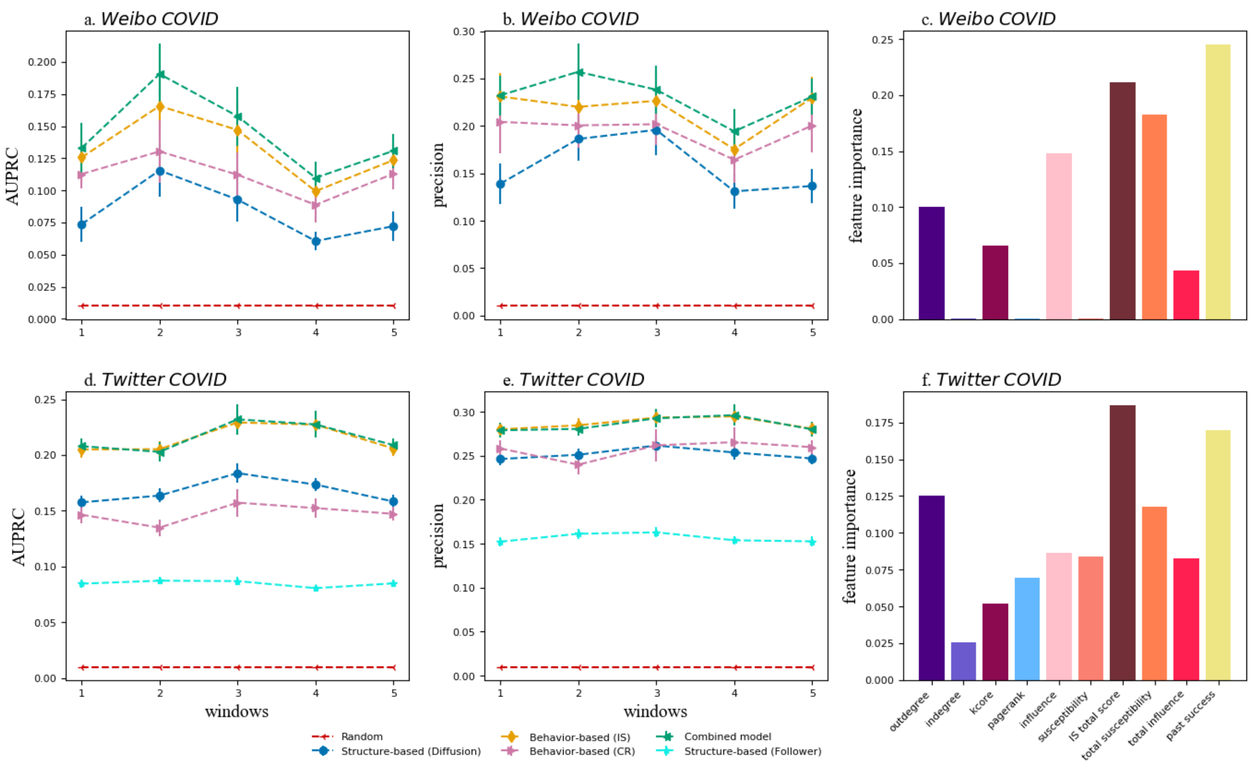

Fig. S28 Predicting superspreaders. We employ the random forest classification algorithm and use different features as input to predict superspreaders, where the superspreaders are defined as individuals with top 1% spreading capacity. Panels **a, b, d, e** show the superspreaders predicting performance on Weibo COVID and Twitter COVID. Two metrics, AURPC, and precision, are adopted to measure the performance of models. Across all windows, the best-performing model is either the combined model or the Behavior-based (IS) model, which points to the essential role of the IS scores for the superspreader prediction. Panels **e, f, g, h** show the feature importance obtained from training the combined model. IS-based features tend to be more important than centrality-based features.

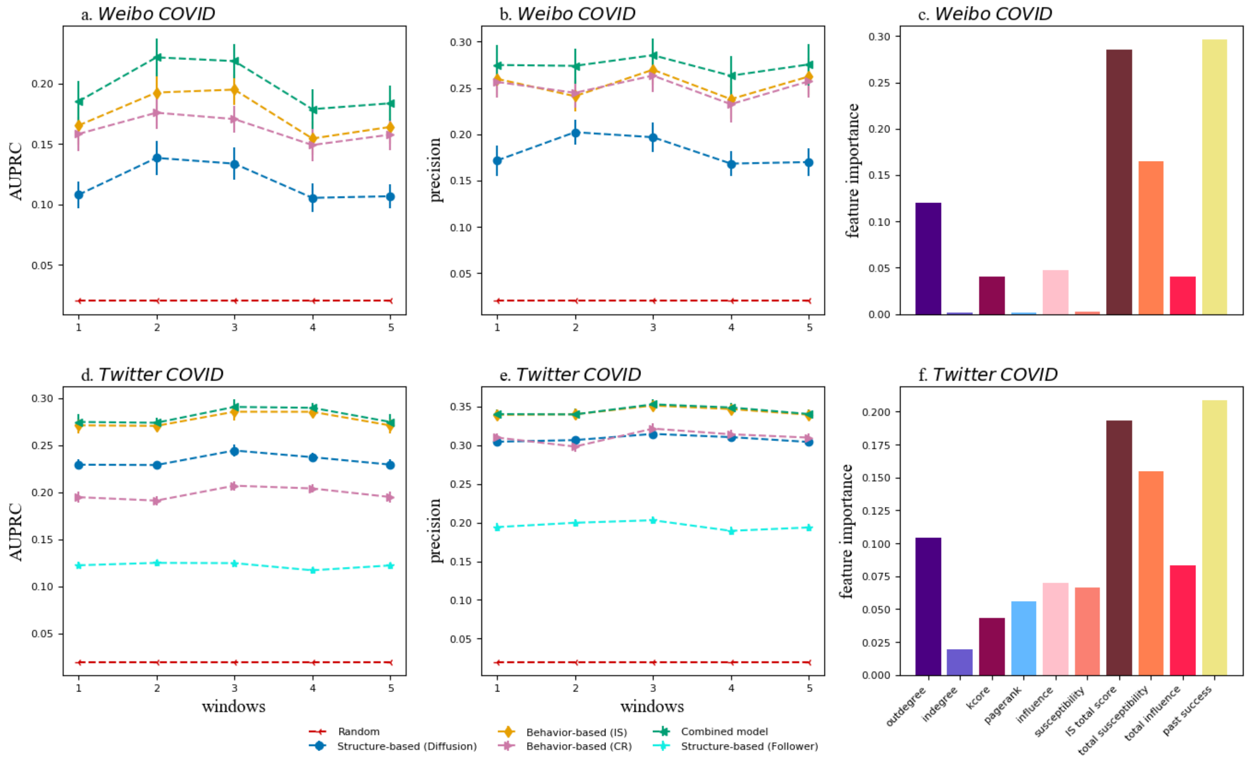

Fig. S29 Predicting superspreaders. We employ the random forest classification algorithm and use different features as input to predict superspreaders, where the superspreaders are defined as individuals with top 2% spreading capacity. Panels **a, b, d, e** show the superspreaders predicting performance on Weibo COVID and Twitter COVID. Two metrics, AURPC, and precision, are adopted to measure the performance of models. Panels **c, f** show the feature importance resulting from training the combined model. Across all windows, the best-performing model is either the combined model or the Behavior-based (IS) model, which points to the essential role of the IS scores for the superspreader prediction. Panels **c, f** show the feature importance obtained from training the combined model. IS-based features tend to be more important than centrality-based features.

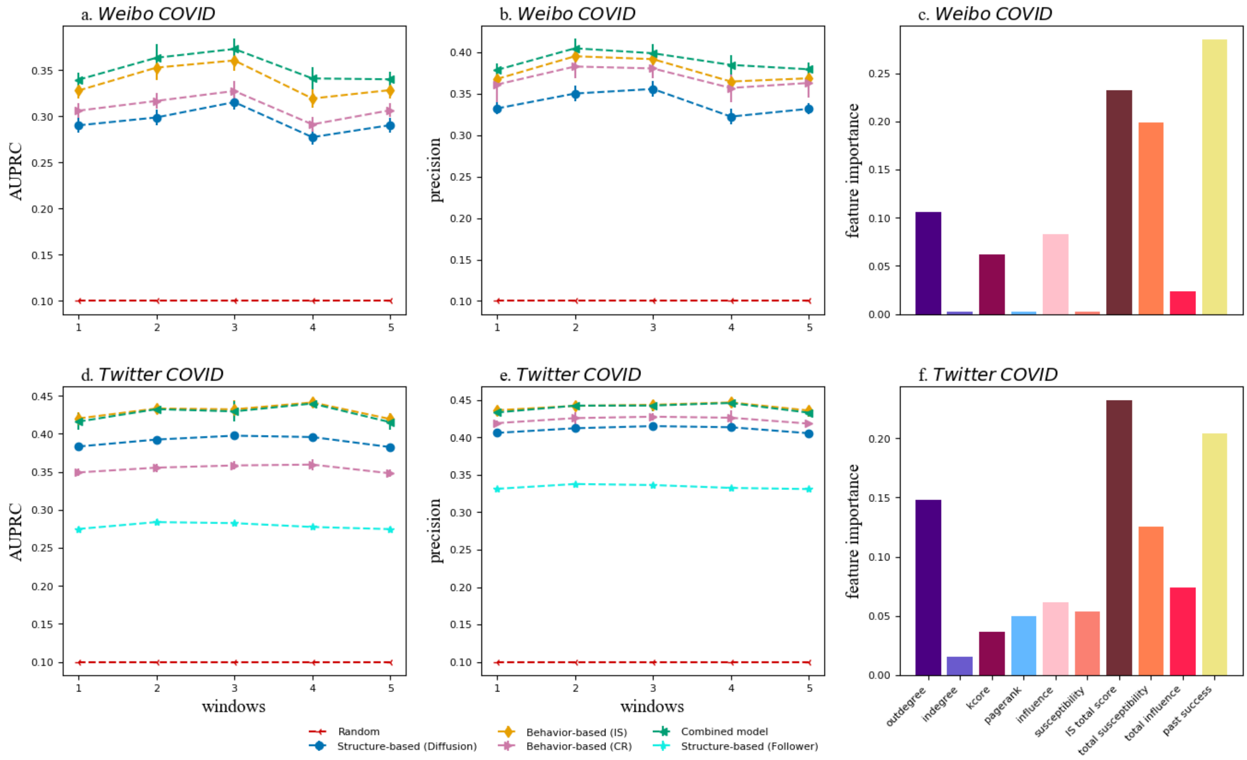

Fig. S30 Predicting superspreaders. We employ the random forest classification algorithm and use different features as input to predict superspreaders, where the superspreaders are defined as individuals with top 10% spreading capacity. Panels **a, b, d, e** show the superspreaders predicting performance on Weibo COVID and Twitter COVID. Two metrics, AURPC, and precision, are adopted to measure the performance of models. Panels **c, f** show the feature importance resulting from training the combined model. Across all windows, the best-performing model is either the combined model or the Behavior-based (IS) model, which points to the essential role of the IS scores for the superspreader prediction. Panels **c, f** show the feature importance obtained from training the combined model. IS-based features tend to be more important than centrality-based features.

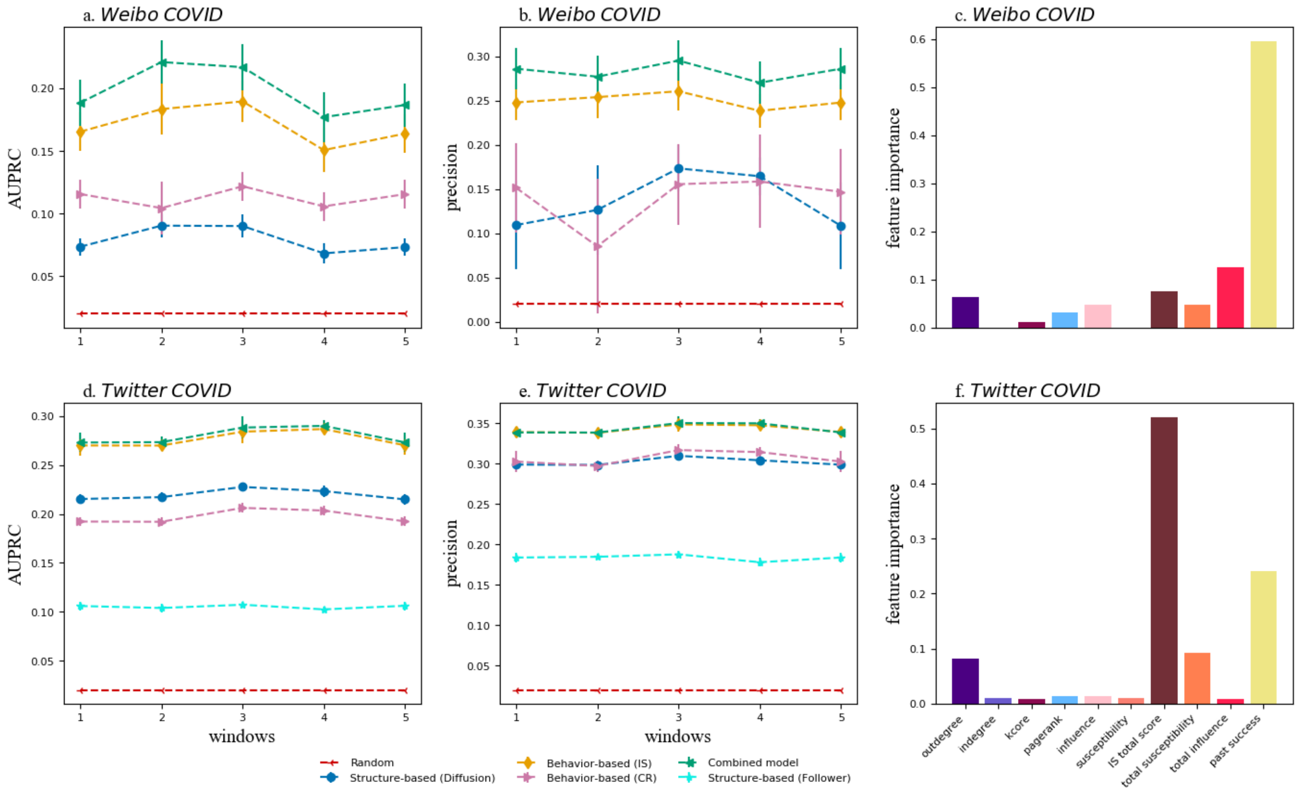

Fig. S31 Predicting superspreaders. We employ the XGBoost classification algorithm and use different features as input to predict superspreaders, where the superspreaders are defined as individuals with top 2% spreading capacity. Panels **a, b, d, e** show the superspreaders predicting performance on Weibo COVID and Twitter COVID. Two metrics, AURPC, and precision, are adopted to measure the performance of models. Panels **c, f** show the feature importance resulting from training the combined model. IS-based features tend to be more important than centrality-based features. Across all windows, the best-performing model is either the combined model or the Behavior-based (IS) model, which points to the essential role of the IS scores for the superspreader prediction. Panels **c, f** show the feature importance obtained from training the combined model. IS-based features tend to be more important than centrality-based features.

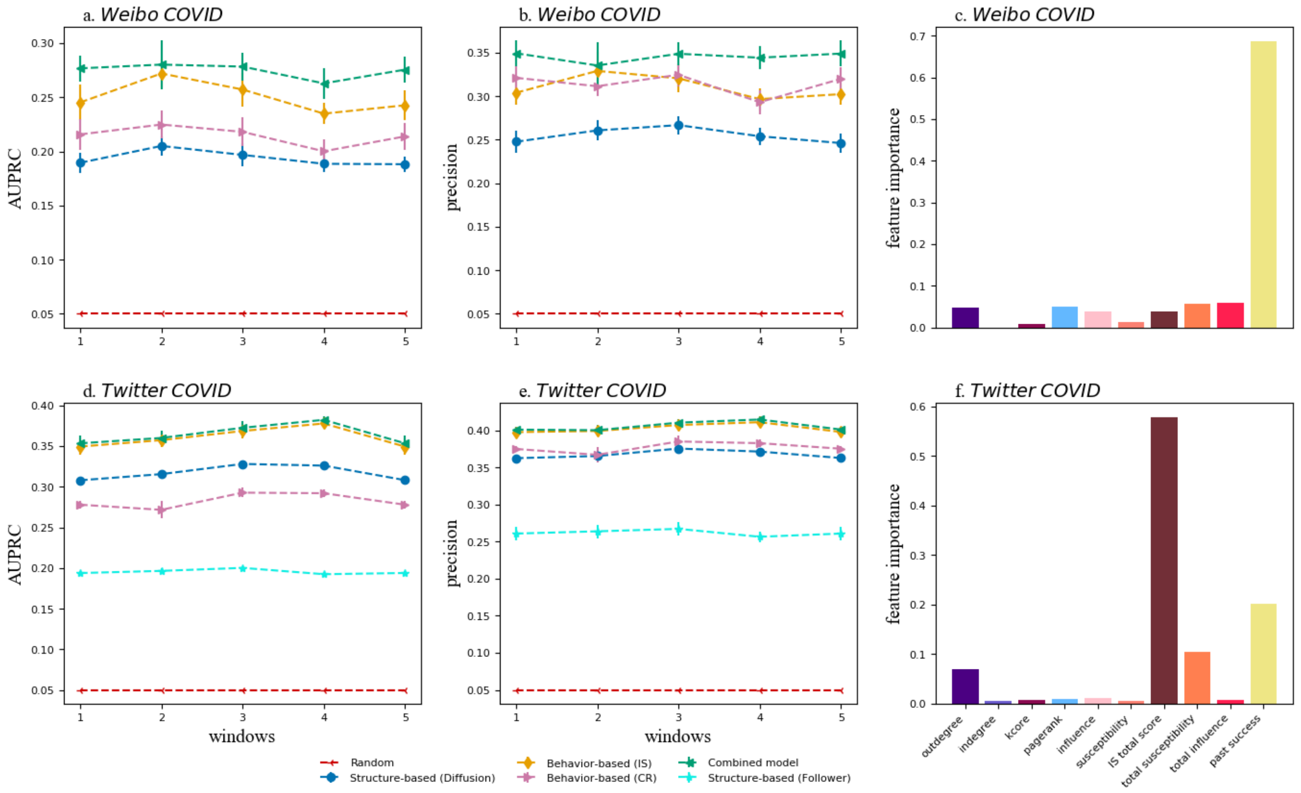

Fig. S32 Predicting superspreaders. We employ the XGBoost classification algorithm and use different features as input to predict superspreaders, where the superspreaders are defined as individuals with top 5% spreading capacity. Panels **a, b, d, e** show the superspreaders predicting performance on Weibo COVID and Twitter COVID. Two metrics, AURPC and precision, are adopted to measure the performance of models. Panels **c, f** show the feature importance resulting from training the combined model. IS-based features tend to be more important than centrality-based features. Across all windows, the best-performing model is either the combined model or the Behavior-based (IS) model, which points to the essential role of the IS scores for the superspreader prediction. Panels **c, f** show the feature importance obtained from training the combined model. IS-based features tend to be more important than centrality-based features.

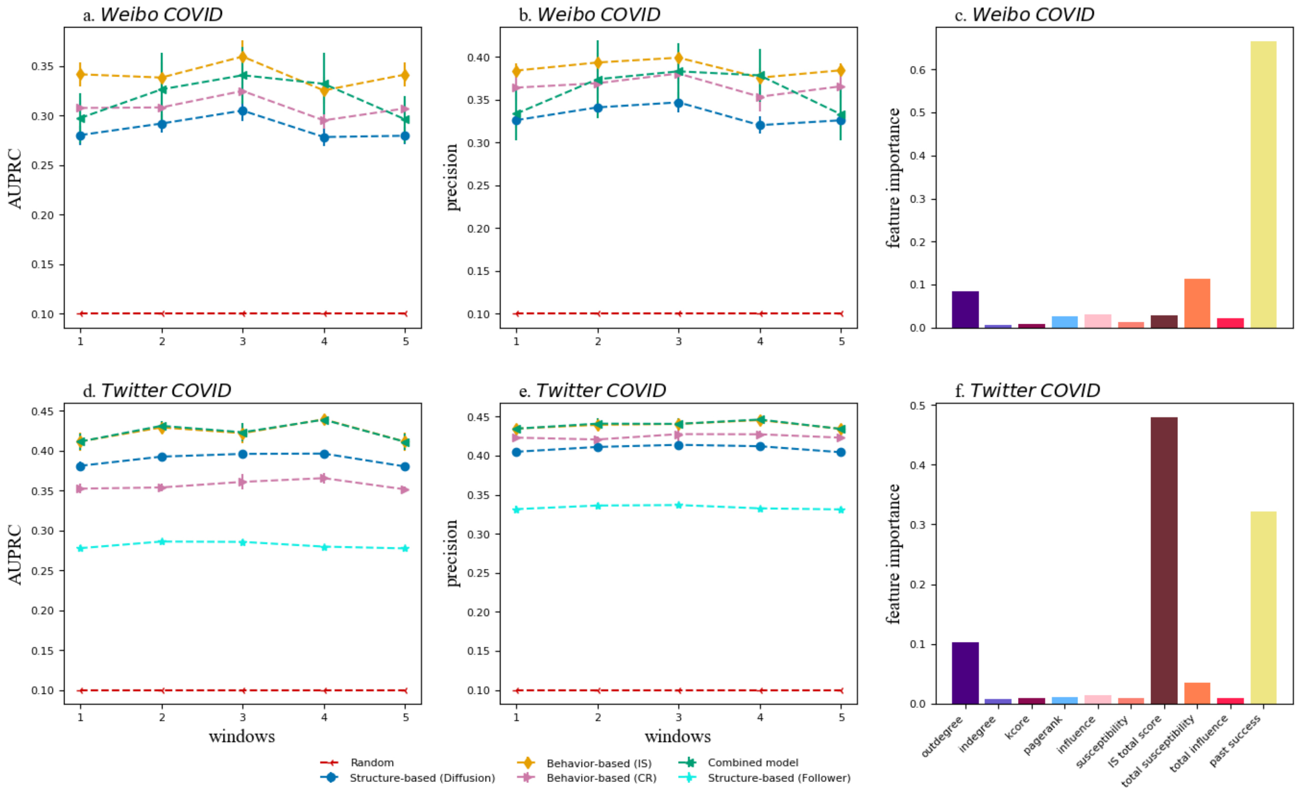

Fig. S33 Predicting superspreaders. We employ the XGBoost classification algorithm and use different features as input to predict superspreaders, where the superspreaders are defined as individuals with top 10% spreading capacity. Panels **a, b, d, e** show the superspreaders predicting performance on Weibo COVID and Twitter COVID. Two metrics, AURPC and precision, are adopted to measure the performance of models. Panels **c, f** show the feature importance resulting from training the combined model. IS-based features tend to be more important than centrality-based features. Across all windows, the best-performing model is either the combined model or the Behavior-based (IS) model, which points to the essential role of the IS scores for the superspreader prediction. Panels **c, f** show the feature importance obtained from training the combined model. IS-based features tend to be more important than centrality-based features.

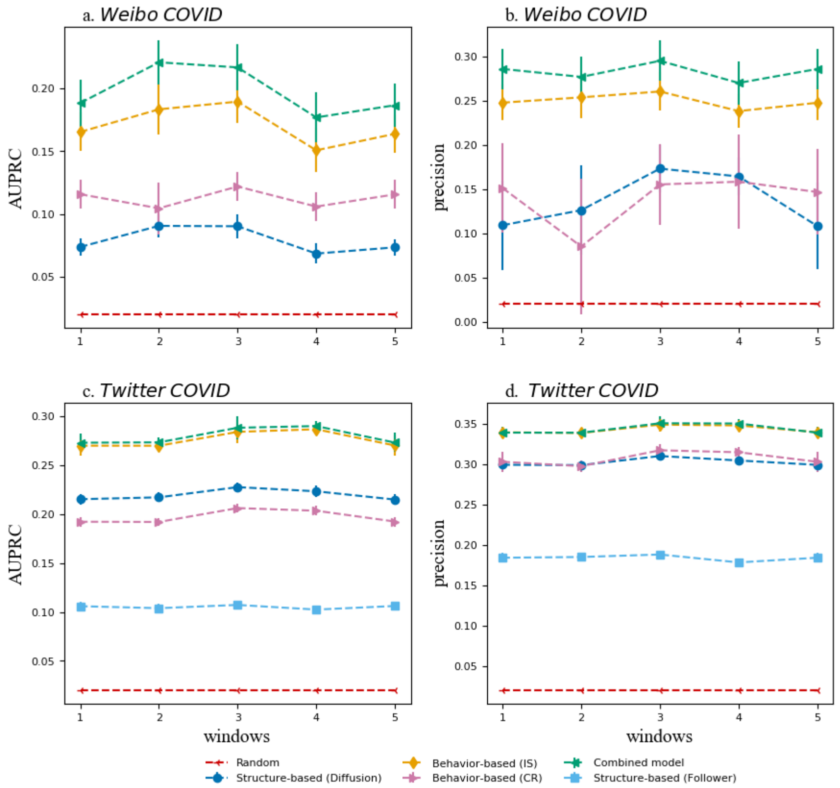

Fig. S34 Predicting superspreaders. We employ the MLP algorithm and use different features as input to predict superspreaders, where the superspreaders are defined as individuals with top 2% spreading capacity. Panels **a, b, c, d** show the superspreaders predicting performance on Weibo COVID and Twitter COVID. Two metrics, AURPC and precision, are adopted to measure the performance of models. Taking into account individuals' behavior-based (IS) features can improve the predicting performance of the model significantly. Across all windows, the best-performing model is either the combined model or the Behavior-based (IS) model, which points to the essential role of the IS scores for the superspreader prediction.

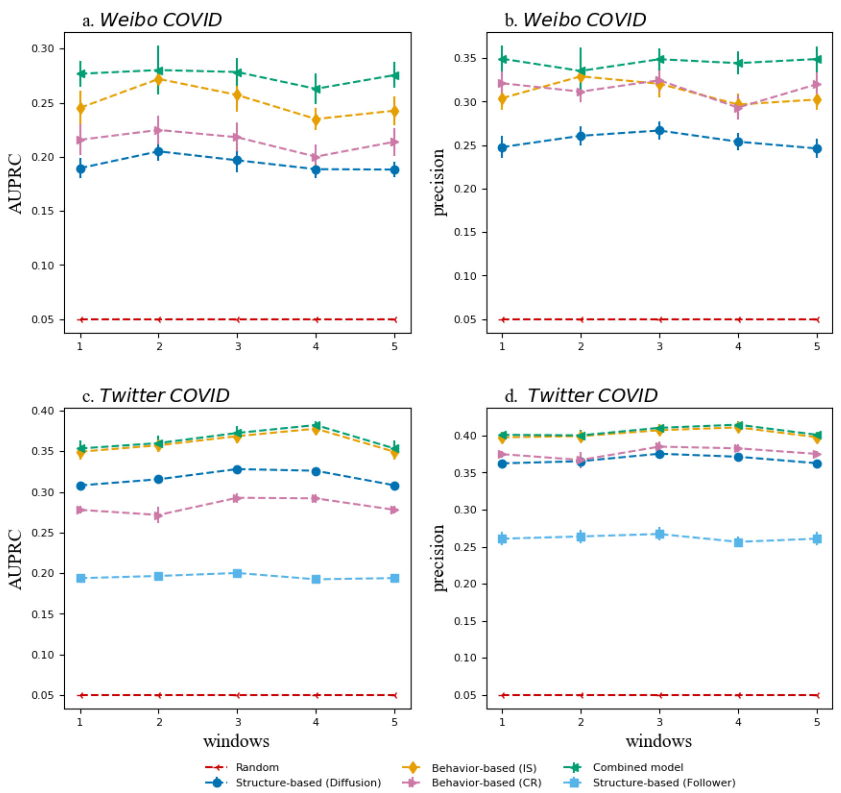

Fig. S35 Predicting superspreaders. We employ the MLP algorithm and use different features as input to predict superspreaders, where the superspreaders are defined as individuals with top 5% spreading capacity. Panels **a, b, c, d** show the superspreaders predicting performance on Weibo COVID and Twitter COVID. Two metrics, AURPC and precision, are adopted to measure the performance of models. Across all windows, the best-performing model is either the combined model or the Behavior-based (IS) model, which points to the essential role of the IS scores for the superspreader prediction.

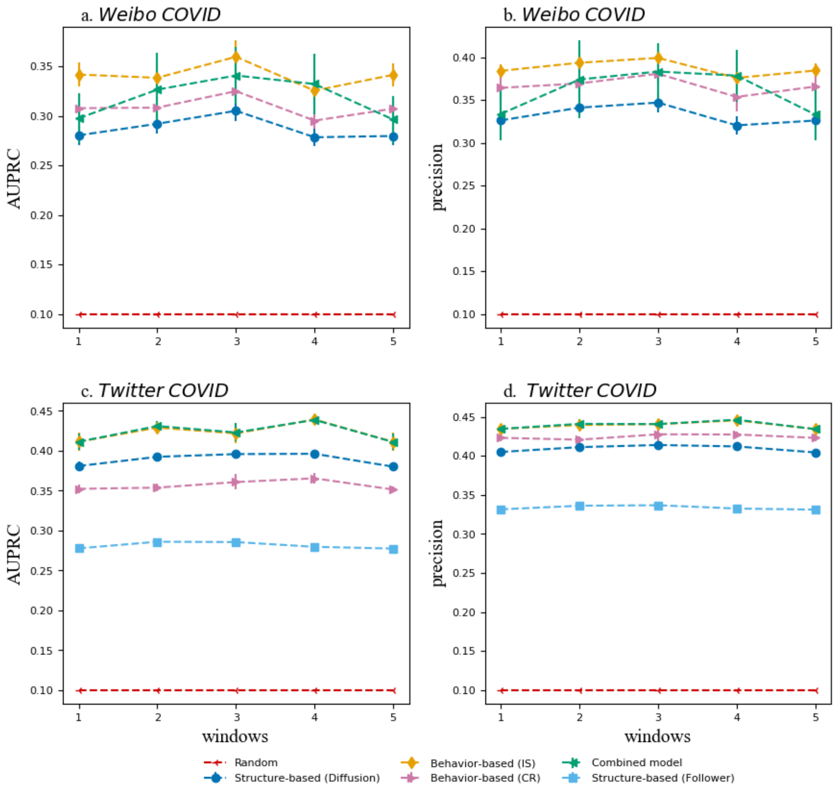

Fig. S36 Predicting superspreaders. We employ the MLP algorithm and use different features as input to predict superspreaders, where the superspreaders are defined as individuals with top 10% spreading capacity. Panels **a, b, c, d** show the superspreaders predicting performance on Weibo COVID and Twitter COVID. Two metrics, AURPC and precision, are adopted to measure the performance of models. Across all windows, the best-performing model is either the combined model or the Behavior-based (IS) model, which points to the essential role of the IS scores for the superspreader prediction.

References

1. Aral, S. & Walker, D. Identifying influential and susceptible members of social networks. *Science* **337**, 337–341 (2012).
2. Leskovec, J., Kleinberg, J. & Faloutsos, C. Graph evolution: Densification and shrinking diameters. *ACM Trans Knowl Discov Data* **1**, 2–es (2007).
3. Zhou, F., Lü, L. & Mariani, M. S. Fast influencers in complex networks. *Commun Nonlinear Sci Numer Simul* **74**, 69–83 (2019).
4. McAuley, J. J. & Leskovec, J. Learning to discover social circles in ego networks. In *Advances in Neural Information Processing Systems*, 539–547 (2012).
5. Aral, S. & Dhillon, P. S. Social influence maximization under empirical influence models. *Nat Hum Behav* **2**, 375–382 (2018).
6. Pei, S., Muchnik, L., Andrade Jr, J. S., Zheng, Z. & Makse, H. A. Searching for superspreaders of information in real-world social media. *Sci Rep* **4**, 1–12 (2014).
7. Newman, M. E., Strogatz, S. H. & Watts, D. J. Random graphs with arbitrary degree distributions and their applications. *Phys Rev E* **64**, 026118 (2001).
8. Servedio, V. D., Buttà, P., Mazzilli, D., Tacchella, A. & Pietronero, L. A new and stable estimation method of country economic fitness and product complexity. *Entropy* **20**, 783 (2018).
9. Gerschgorin, S. Uber die abgrenzung der eigenwerte einer matrix. *Her Russ Acad Sci* **7**, 749–754 (1931).
10. Baltagi, B. H. & Baltagi, B. H. *Econometric analysis of panel data*, vol. 4 (New York: Springer, 2008).
11. Breiman, L. Random forests. *Mach Learn* **45**, 5–32 (2001).
12. Chen, T. & Guestrin, C. Xgboost: A scalable tree boosting system. In *Proceedings of the 22nd acm sigkdd international conference on knowledge discovery and data mining*, 785–794 (2016).
13. Gardner, M. W. & Dorling, S. Artificial neural networks (the multilayer perceptron)—a review of applications in the atmospheric sciences. *Atmos Environ* **32**, 2627–2636 (1998).
14. Banda, J. M. *et al.* A large-scale COVID-19 Twitter chatter dataset for open scientific research - an international collaboration (2020).
15. Ratkiewicz, J., Fortunato, S., Flammini, A., Menczer, F. & Vespignani, A. Characterizing and modeling the dynamics of online popularity. *Phys Rev Lett* **105**, 158701 (2010).
16. Tacchella, A., Cristelli, M., Caldarelli, G., Gabrielli, A. & Pietronero, L. A new metrics for countries' fitness and products' complexity. *Sci Rep* **2**, 1–7 (2012).
17. Mazzilli, D., Mariani, M. S., Morone, F. & Patelli, A. Fitness in the light of sinkhorn. arXiv:2212.12356 .
18. Marshall, A. W. & Olkin, I. Scaling of matrices to achieve specified row and column sums. *Numer Math* **12**, 83–90 (1968).
19. Silverman, B. W. *Density estimation for statistics and data analysis* (New York: Routledge, 2018).
